# Platelets induce increased estrogen production through NF-κB and TGF-β1 signaling pathways in endometriotic stromal cells

**DOI:** 10.1038/s41598-020-57997-6

**Published:** 2020-01-28

**Authors:** Qiuming Qi, Xishi Liu, Qi Zhang, Sun-Wei Guo

**Affiliations:** 1 Shanghai OB/GYN Hospital, Fudan University, Shanghai, 200011 China; 2 Shanghai Key Laboratory of Female Reproductive Endocrine-Related Diseases, Fudan University, Shanghai, 200011 China

**Keywords:** Transcription, Endocrine reproductive disorders

## Abstract

Endometriosis is estrogen-dependent disorder. Two theories provide the explanations for the increased estrogen production. One is the feed-forward loop model linking inflammation and estrogen production. The more recent model evokes the tissue hypoxia resulting from endometrial debris detached and then regurgitated to the peritoneal cavity. Both models tacitly assume that everything occurs within the endometriotic stromal cells, seemingly without the need for exogenous factors. This study was undertaken to investigate as whether platelets may be responsible for local estrogen overproduction. We employed *in vitro* experimentation that evaluated the 17β-estradiol (E_2_) levels in endometriotic stromal cells treated with activated platelets, and the genes and protein expression levels of StAR, HSD3B2, aromatase, and HSD17B1, as well as their upstream genes/proteins such as NF-κB, TGF-β1, HIF-1α, SF-1 and phosphorylated CREB. In addition, we conducted 2 animal experimentations using platelet depletion/infusion and also neutralization of NF-κB and TGF-β1, followed by immunohistochemistry analysis of involved in StAR, HSD3B2, aromatase, and HSD17B1, as well as SF-1 and p-CREB. We found that treatment of endometriotic stromal cells by activated platelets increase the E_2_ production by 4.5 fold, and concomitant with increased gene and protein expression of StAR, HSD3B2, aromatase, and HSD17B1, the four genes/enzymes important to estrogen synthesis, along with their upstream genes HIF-1α, SF-1 and phosphorylated CREB. Moreover, platelets activate these genes through the activation of NF-κB and/or TGF-β1, and antagonism of either signaling pathway can abolish the induction of the 4 genes and thus increased estrogen production. The two animal experimentations confirmed these changes. Thus, platelets increase the E_2_ production in endometriotic stromal cells through upregulation of StAR, HSD3B2, aromatase, and HSD17B1 via the activation of NF-κB and/or TGF-β1. These findings provide a yet another compelling piece of evidence that endometriotic lesions are indeed wounds undergoing repeated tissue injury and repair. They strongly indicate that non-hormonal therapeutics for endometriosis is theoretically viable, with anti-platelet therapy being one promising avenue.

## Introduction

Endometriosis, defined as the deposition and growth of endometrium-like tissues outside the uterine cavity, is a common disorder affecting about 6–10% of women of reproductive age^1^. As a major contributor to pelvic pain and infertility^2^ impacting negatively on women’s quality of life^3^, a source of anxiety and depression^4^ and a leading cause of gynecological hospitalization in the United States^5^ and likely in many other parts of the world, endometriosis is frequently viewed as an enigmatic disease due to its elusive pathogenesis and pathophysiology^6,7^. Consequently, its effective treatment remains a challenge^2,8^.

Endometriosis has been viewed as a ultimate hormonal disease^9^, featuring estrogen-dependent growth and maintenance of ectopic endometrium as well as increased local production of estrogens due to molecular aberrations in steroidogenesis^10^. It is also commonly viewed as a pelvic inflammatory condition, featuring increased production of proinflammatory cytokines and chemokines^11^. So far perhaps the most comprehensive model that encompasses various mechanisms underlying both elevated 17β-estradiol (E_2_) production and increased of proinflammatory cytokines and chemokines is the feed-forward model proposed by Bulun *et al*.^10^, in which proinflammatory cytokines activate cyclooxygenase-2 (COX-2), resulting in increased production of prostaglandin E_2_ (PGE_2_), which, in turn, stimulates some key genes involved in the production of E_2_, such as steroidogenic acute regulatory protein (StAR), aromatase and 17β-hydroxysteroid dehydrogenase type-1 (HSD17B1), resulting in elevated production of E_2_, the most potent estrogen. The increased E_2_ further induces estrogen receptor β (ERβ), yielding further induction of COX-2. This positive feedback process, once started, supposedly perpetuates if untamed, resulting in increased inflammation due to elevated PGE_2_ levels and increased growth because of potent mitogenic effect of E_2_^10^.

PGE_2_ activates the protein kinase A (PKA) signaling pathway via raising the intracellular levels of cyclic adenosine 3′,5′-monophosphate (cAMP)^12–14^, which can enhance the binding of steroidogenic factor-1 (SF-1) to promoters of these steroidogenic genes^15^, and induce phosphorylation of the transcriptional activator cAMP-response element-binding protein (CREB)^16^. The binding of SF-1 and CREB to the promoters of steroidogenic genes is responsible for inducing the expression levels and activity of these enzymes, thus promoting the estrogen biosynthesis in endometriotic stromal cells^10,17,18^.

One salient feature of this positive-feedback loop between inflammation and estrogen production in endometriosis is that everything occurs within the endometriotic lesions, or, more precisely, endometriotic stromal cells, seemingly without the need for extraneous factors. However, just as “it takes a village to raise a child”, an endometriotic lesion does not come into being completely on its own. Rather, its development and the subsequent manifestation of various symptomology require, by necessity, aid and assistance from many aiders and abettors. Indeed, accumulating evidence suggests that endometriotic lesions are essentially wounds undergoing repeated tissue injury and repair (ReTIAR)^19–22^, and, as such, many non-endometriotic cells in the lesional microenvironment, such as platelets^21,23^, macrophages^19,24^, and now nerve fibers^25,26^, are actively involved in facilitating lesional development.

In wound healing, estrogen has been well documented to be actively involved^27,28^. Numerous studies have shown that estrogen is important to wound healing, and its deficiency delays or impairs wound healing^29–33^. In fact, estrogen is found to be involved in all phases of wound healing^27^. One gene expression profiling study of wound tissues from young and elder men found that among genes that were differentially expressed, 78% of them being estrogen-regulated and only 3% being age-related^34^, suggesting that estrogen is more important than intrinsic aging in wound healing. Remarkably, in striking similarity to endometriotic lesions in which ERβ is shown to be overexpressed^35,36^, ERβ has been shown to play a critical role in wound healing^37,38^.

These findings in wound healing raise a question as whether culprits other than endometriotic cells in the lesional microenvironment could also be responsible for the increased local estrogen production. Recent studies have shown that hypoxia, occurred to menstrual debris that is devoid of blood supply when regurgitated into the peritoneal cavity, can dramatically turn endometrial stromal cells into endometriotic counterpart, causing completely different phenotypes^39^. This notion may explain as why endometrial debris may invade and then establish itself in ectopic sites. Since platelets are the first responder to injury, we wondered as whether activated platelets can induce a hypoxic state and indeed, we recent found that platelets can induce hypoxia-inducible factor 1α (HIF-1α) expression, effectively inducing a hypoxic sate in both endometriotic and endometrial stromal cells^40^. Thus, we hypothesized that activated platelets may activate genes involved in estrogen biosynthesis, resulting in increased local estrogen levels. This study was undertaken to test this hypothesis.

## Results

### Activated platelets induce increased E_2_ production in endometriotic stromal cells

**Figure 1:**
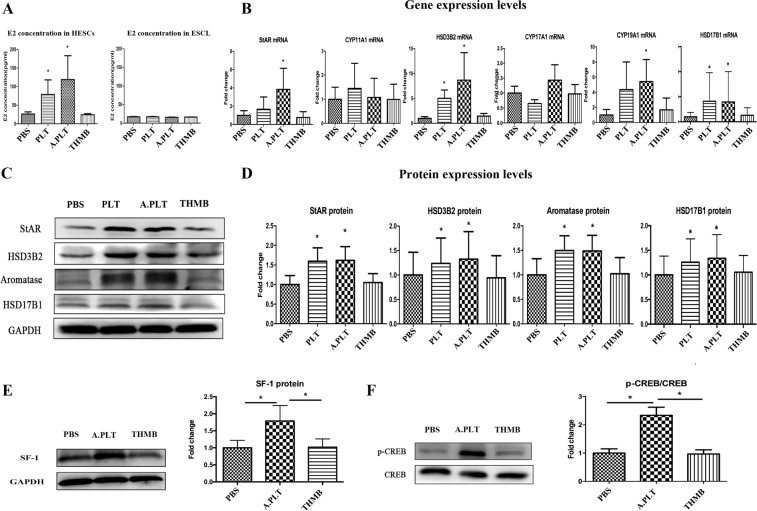
Treatment of HESCs with activated platelets resulted in increased production of E_2_ through upregulation of steroidogenic genes. (**A**) Concentrations of E_2_ in the supernatant of HESCs (n = 7) and ESCL (n = 5) treated with PBS, platelets, activated platelets, and thrombin alone. Gene (**B**) and protein (**D**) expression levels of the genes involved in estrogen biosynthesis: StAR, CYP11A1, HSD3B2, CYP17A1, CYP19A1 and HSD17B1 in HESCs co-cultured with PBS, platelets, activated platelets and thrombin alone. (**C**) Representative Western blotting results for StAR, HSD3B2, aromatase and HSD17B1. Fold change for SF-1 (**E**) and p-CREB (**F**) protein expression in HESCs co-cultured with PBS, activated platelets and thrombin alone. “*” denotes that the p-value of the difference between the designated treatment and the PBS treatment is less than 0.05. Abbreviations used: PLT: platelets; A.PLT: activated platelets; THMB: thrombin. Paired Wilcoxon’s test was used.

To see whether activated platelets induce E_2_ production in endometriotic stromal cells, we first measured E_2_ concentration in the supernatant in human endometriotic stromal cells (HESCs) co-cultured for 48 hours with PBS, platelets alone, activated platelets, or thrombin alone. We found that, compared with those treated with PBS, the E_2_ concentration was significantly elevated in HESCs co-cultured with either platelets or activated platelets (both p-values = 0.018), but with thrombin alone (p = 0.87, Fig. 1A, left panel). Notably, HESCs co-cultured with platelets and activated platelets produced E_2_ that was 3.0 and 4.5 fold, respectively, higher than that treated with PBS (79.15 pg/mL and 119.16 pg/mL, respectively, vs. 26.32 pg/mL). In contrast, the E_2_ concentrations were all very low in the normal endometrial stromal cell line (ESCL), irrespective co-culture with or without activated platelets (Fig. 1A, right panel). To rule out the possibility that the E_2_ was synthesized by activated platelets, we also evaluated, in the absence of endometriotic or endometrial cells, the E_2_ concentration in the medium that was cultured with or without activated platelets for 48 hours and only found barely detectable E_2_ in all groups (data not shown), suggesting that the increased E_2_ came indeed from endometrial/endometriotic stromal cells only. The difference in E_2_ production between HESCs and ESCLs is consistent with what have been documented previously^41–45^.

### Activated platelets upregulate key genes involved in estrogen biosynthesis in endometriotic stromal cells

Given increased E_2_ production in endometriotic stromal cells stimulated by activated platelets, we next evaluated the gene and protein expression levels in key genes critically involved in estrogen biosynthesis. Consistently, the mRNA levels of StAR, HSD3B2, aromatase and HSD17B1, but not CYP11A1 or CYP17A1, in HESC cells co-cultured with activated platelets, but not thrombin alone (all p’s > 0.20), were all significantly higher than those treated with vehicle (all p’s ≤ 0.028, Fig. 1B). The protein levels of StAR, HSD3B2, aromatase and HSD17B1 in HESCs co-cultured with platelets or activated platelets, but not thrombin alone, were also significantly higher than that that were co-cultured with vehicle (all p’s ≤ 0.017, Fig. 1C,D). Interestingly, the activated platelets increased the protein expression levels of StAR, HSD3B2, aromatase, and HSD17B1 by an average of 1.6, 1.3, 1.5, and 1.3 fold, respectively (Fig. 1C,D), and their product, i.e., 1.6 × 1.3 × 1.5 × 1.3 = 4.1, is uncannily close to 4.5 fold increase in E_2_ production as shown above.

To see whether platelets also affected the genes upstream of StAR, HSD3B2, aromatase and HSD17B1, we also evaluated the protein expression levels of SF-1 and p-CREB in HESCs in different co-culture conditions. We found that activated platelets, but not thrombin alone, resulted in significantly higher SF-1 and p-CREB protein expression levels as compared with controls (all p-values = 0.012, Fig. 1E,F), suggesting that activated platelets acted on the SF-1/CREB level, at least, and possibly higher level, in the E_2_ production.

### Suppression of NF-κB activation abolishes platelet-induced upregulation of HIF-1α, COX-2, and VEGF

**Figure 2:**
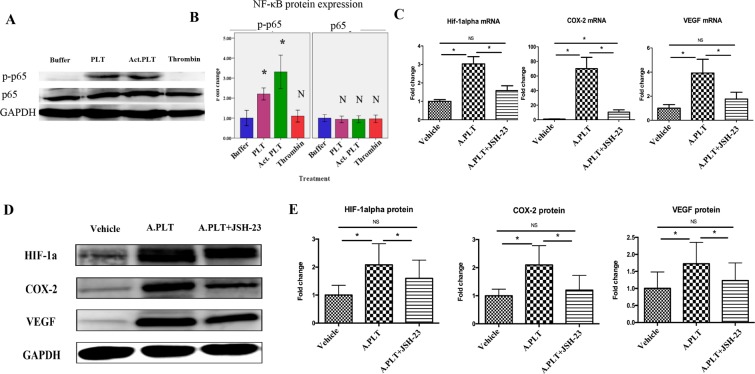
Activated platelets activate NF-κB, and suppression of NF-κB activation abolishes platelet-induced upregulation of HIF-1α, COX-2, and VEGF. (**A**) Representative Western blotting results of phorphorylated-p65 in HESCs (n = 8) co-cultured with PBS (buffer), platelets, activated platelets, and thrombin. (**B**) Summary of Western blot results. (**C**) Fold change for HIF-1α, COX-2 and VEGF mRNA abundance in HESCs (n = 8) co-cultured with vehicle, activated platelets and activated platelets + JSH-23. (**D**) Representative Western blotting results and fold change for HIF-1α, COX-2, and VEGF in HESCs co-cultured with vehicle, activated platelets and activated platelets + JSH-23. (**E**) Summary of western blot results. “*” denotes that the p-value of the difference between the groups is less than 0.05. N or NS: p > 0.05. Abbreviation used: A.PLT: activated platelets. Paired Wilcoxon’s test was used.

Given the documented role of prostaglandin E2 (PGE_2_) in estrogen biosynthesis in endometriosis^16,45–47^ and also of hypoxia in turning normal endometrial stromal cells into endometriotic stromal cell-like phenotype^48,49^, we wondered as whether activated platelets could activate NF-κB, the upstream of COX-2, which, in turn, is the gene encoding a rate-limiting enzyme in PGE_2_ production, and also HIF-1α, a key transcription factor that responds to hypoxia^50^, which is reported to be elevated^51^ and can upregulate COX-2 in endometriosis^49^. We found that, indeed, HESCs co-cultured with both platelets and activated platelets increased the protein expression levels of p-p65 (both p < 0.012, Fig. 2A,B). In addition, activated platelets induced significantly higher expression of HIF-1α, COX-2 and VEGF (which is downstream of HIF-1α) at both transcriptional and protein expression levels in endometriotic stromal cells (all p-values ≤ 0.012, Fig. 2C–E), consistent with our previous report^40^. Neutralization of NF-κB by JSH-23 nearly completely abolished this upregulation (all p’s ≤ 0.036, Fig. 2C–E). With the only exception of COX-2 mRNA abundance, which was still significantly higher than that of the HESCs treated with vehicle (p = 0.025, Fig. 2C), the gene and protein expression levels of these genes in HESCs co-cultured with platelets but pre-treated with JSH-23 were no different from controls (all p’s > 0.05, Fig. 2C,E).

### NF-κB neutralization abolishes platelets-induced increase in E_2_ production through downregulation of StAR, HSD3B2, aromatase, HSD17B1, SF-1 and p-CREB in HESCs

**Figure 3:**
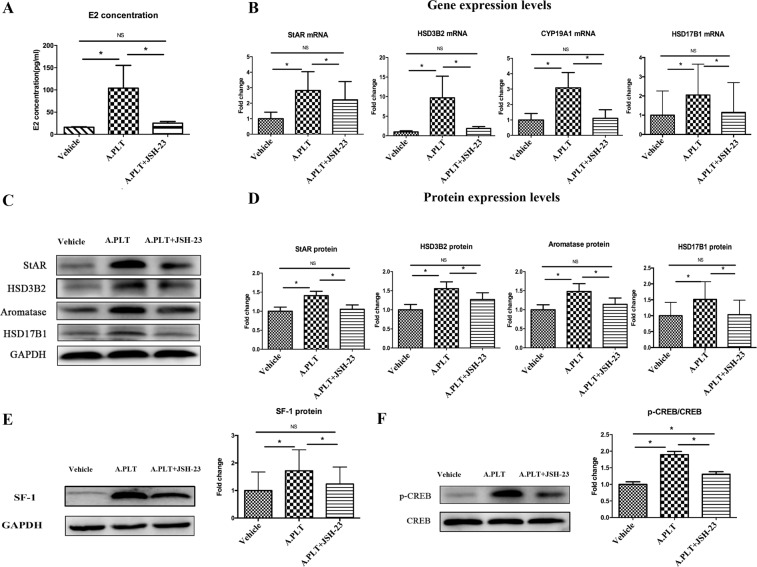
Effect of suppression of NF-κB activation by JSH-23 on the production of E_2_ and expression of steroidogenic genes in HESCs. (**A**) Concentrations of E_2_ in the supernatant of HESCs (n = 8) treated with vehicle, activated platelets and activated platelets + JSH-23. (**B**) Gene expression levels of the genes involved in estrogen biosynthesis StAR, HSD3B2, CYP19A1 and HSD17B1 in HESCs co-cultured with vehicle, activated platelets and activated platelets + JSH-23. (**C**) Representative Western blotting results for StAR, HSD3B2, aromatase and HSD17B1 protein expression. (**D**) Summary of fold changes of protein expression levels of StAR, HSD3B2, aromatase and HSD17B1 in HESCs (n = 8) treated with vehicle, activated platelets and activated platelets + JSH-23. Summary of fold changes for SF-1 (**E**) and p-CREB (**F**) protein expression in HESCs (n = 8) treated with vehicle, activated platelets and activated platelets + JSH-23. “*” denotes that the p-value of the difference between the groups is less than 0.05. NS: p > 0.05. Paired Wilcoxon’s test was used.

Given the above finding that NF-κB neutralization abrogated platelets-induced upregulation of HIF-1α, COX-2, and VEGF, we evaluated the E_2_ levels in the culture medium of HESCs co-cultured with activated platelets with and without pre-treatment with JSH-23. We found that pre-treatment of HESCs with JSH-23 significantly reduced the E_2_ production in culture media as compared with that without the pre-treatment (p = 0.036) without reducing the cell viability (Supplementary Fig. S1), and, in fact, completely abolished the E_2_ production induced by platelets (p = 0.13, Fig. 3A). Consistently, NF-κB neutralization by pre-treatment with JSH-23 completely abolished platelets-induced expression of StAR, HSD3B2, aromatase and HSD17B1 at both transcriptional and protein levels (all p-values ≤ 0.025, Fig. 3B–D). In addition, NF-κB neutralization by pre-treatment with JSH-23 also abolished platelets-induced expression of SF-1 and p-CREB in HESCs, both at the protein levels (both p’s = 0.012, Fig. 3E,F). With the only exception of p-CREB protein levels, the gene and protein expression levels of these genes in HESCs co-cultured with platelets but pre-treated with JSH-23 were no different from controls (all p’s > 0.05, Fig. 3B,D–F).

### TGF-β1 neutralization abolishes platelets-induced upregulation of HIF-1α, COX-2, and VEGF

**Figure 4:**
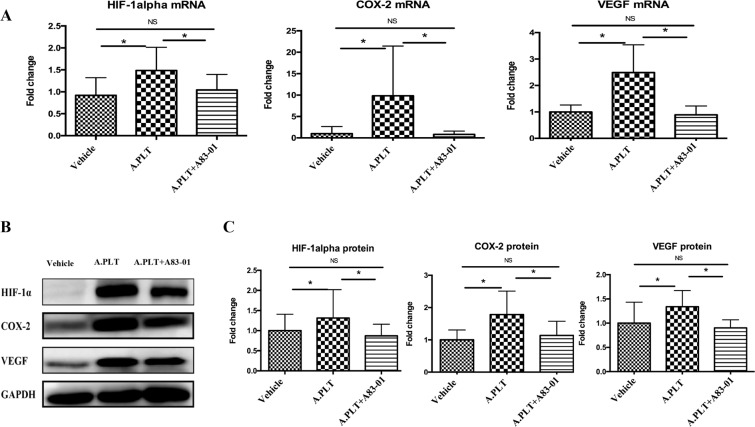
Effect of neutralization of TGF-β1 by A83-01 on platelet-induced gene and protein expression of HIF-1α, COX-2 and VEGF. (**A**) Fold change of HIF-1α, COX-2 and VEGF mRNA abundance in HESCs (n = 8) treated with vehicle, activated platelets and activated platelets + A83-01. (**B**) Representative Western blotting results for HIF-1α, COX-2 and VEGF protein expression in HESCs (n = 8) co-cultured with vehicle, activated platelets and activated platelets + A83-01, and (**C**) Summary of fold changes. “*” denotes that the p-value of the difference between the groups is less than 0.05. NS: p > 0.05. Paired Wilcoxon’s test was used.

We have previously shown that platelet-derived TGF-β1 activates TGF-β1/Smad3 signaling pathway in endometriotic epithelial and stromal cells^21^. We also have shown that activated platelets can induce upregulation of HIF-1α in HESCs^40^. We thus wondered as whether activated platelets could also induce upregulation of COX-2 and VEGF and whether neutralization of TGF-β1 could abrogate such an induction. We found that, at both transcriptional and protein expression levels, the expressions of HIF-1α, COX-2 and VEGF were significantly elevated after co-culture with activated platelets (all p’s ≤ 0.012, Fig. 4A–C), but the upregulation was completely abolished by TGF-β1 neutralization with pre-treatment with A83-01 (all p’s ≤ 0.012, Fig. 4A–C). Thus, TGF-β1 neutralization abolished platelets-induced upregulation of HIF-1α, COX-2, and VEGF in HESCs.

### TGF-β1 neutralization abolishes platelets-induced increase in E_2_ production through downregulation of StAR, HSD3B2, aromatase, HSD17B1, SF-1 and p-CREB in HESCs

**Figure 5:**
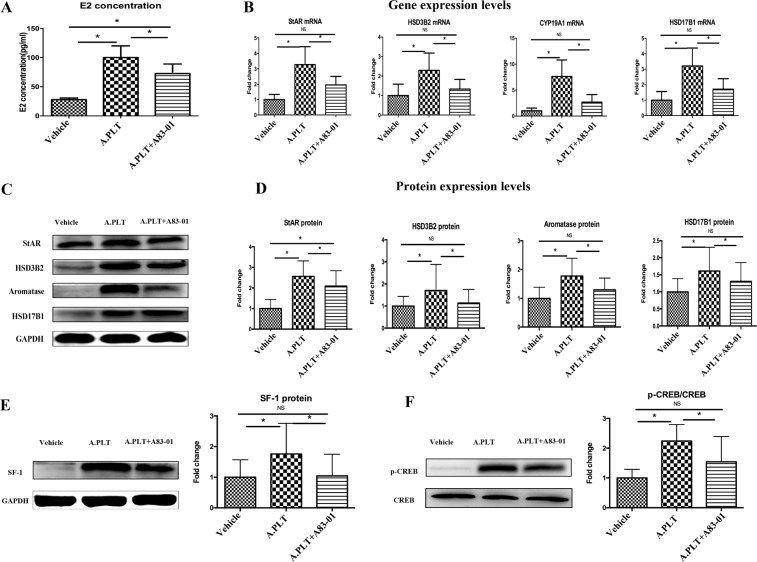
Effects of neutralization of TGF-β1 by A83-01 on the production of E_2_ and expression of genes involved in steroidogenesis in HESCs. (**A**) Concentrations of E_2_ in the supernatant of HESCs (n = 8) treated with vehicle, activated platelets and activated platelets + A83-01. (**B**) Gene expression levels of StAR, HSD3B2, CYP19A1 and HSD17B1 in HESCs (n = 8) treated with vehicle, activated platelets and activated platelets + A83-01. (**C**) Representative Western blotting results for StAR, HSD3B2, aromatase and HSD17B1 in HESCs under different treatments. (**D**) Summary of fold changes of protein expression levels of StAR, HSD3B2, aromatase and HSD17B1 in HESCs (n = 8) treated with vehicle, activated platelets and activated platelets + A83-01. Western blot analysis results for SF-1 (**E**) and p-CREB (**F**) proteins in HESCs treated with vehicle, activated platelets and activated platelets + A83-01. “*” denotes that the p-value of the difference between the groups is less than 0.05. NS: p > 0.05. Paired Wilcoxon’s test was used.

Next, we evaluated the E_2_ levels in HESCs co-cultured with activated platelets with and without TGF-β1 neutralization through pre-treatment with A83-01 or vehicle. We found that pre-treatment of HESCs with A83-01 significantly reduced the E_2_ production in culture media as compared with that without the pre-treatment (p = 0.017) without reducing the cell viability (Supplementary Fig. S1), but the resultant E_2_ levels were still significantly higher than those pre-treated with vehicle (p = 0.012, Fig. 5A). Consistently, TGF-β1 neutralization by pre-treatment with A83-01 completely abolished platelets-induced expression of StAR, HSD3B2, aromatase and HSD17B1 at both transcriptional and protein levels (all p’s ≤ 0.025), with the only exception of the StAR protein levels which were still significantly higher than the control (p = 0.012, Fig. 5B–D). In addition, TGF-β1 neutralization by pre-treatment with A83-01 also completely abolished platelets-induced expression of SF-1 and p-CREB in HESCs, both at the protein levels (both p’s ≤ 0.05, Fig. 5E,F).

### Platelet infusion increases, while platelet depletion reduces, the expression of steroidogenic proteins in endometriotic lesions in mice with induced endometriosis

**Figure 6:**
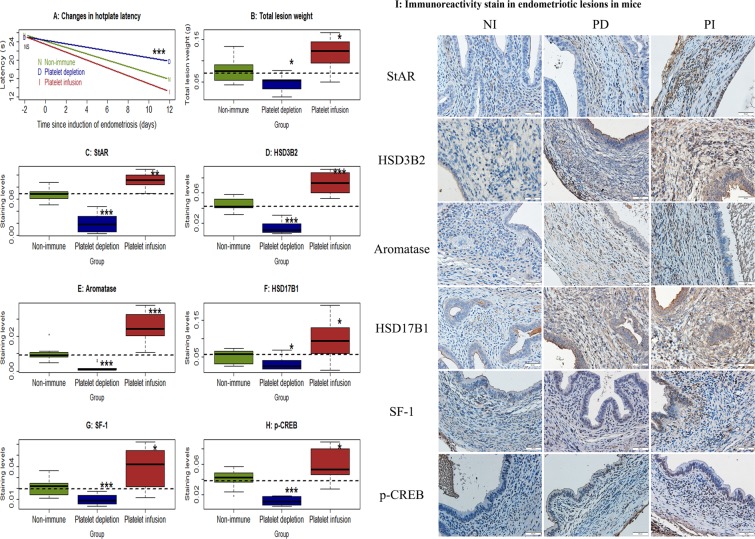
Effect of non-immune serum (NI), platelet infusion (PI) and platelet depletion (PD) on lesional development and lesional expression of steroidogenic proteins in mice with induced endometriosis. (**A**) Change in hotplate latency for mice in the NI, PD and PI groups tested at the time indicated. (**B**) Total lesion weight for mice in the 3 treatment groups. Boxplots of lesional staining levels in NI, PD and PI groups: StAR (**C**), HSD3B2 (**D**), aromatase (**E**), HSD17B1 (**F**), SF-1 (**G**), and p-CREB (**H**). The dotted horizontal line represents the median of all 3 groups. Symbols of statistical significance: *: p < 0.05, **:p < 0.01, ***: p < 0.001. n = 10 for each group. Except in (**A**), where the comparison was made using Kruskal’s test, all tests were made using Wilcoxon’s test in comparison with the NI group. (**I**) Representative photomicrographs of StAR, HSD3B2, aromatase, HSD17B1, SF-1 and p-CREB staining in endometriotic lesions (×400) in Balb/c mice with induced endometriosis in different treatment groups. Scale bar = 50 μm.

To further confirm the role of platelets in regulating genes involved in estrogen biosynthesis in endometriotic lesions, we carried out a mouse experiment using platelet depletion (PD) and platelet infusion (PI). We found that, while there was no difference in hotplate latency among the 3 groups of mice 2 days prior to the induction of endometriosis (p = 0.98, Fig. 6A), but 12 days after the induction the difference was highly significant (p = 0.0001, Fig. 6A). In particular, PI mice had significantly shorter (p = 0.018), while PD mice had significantly longer (p = 0.0055), latency than the control mice injected with a matched non-immune (NI) antibody. In addition, compared with the NI mice that received a mock antibody, the total lesion weight in PI mice increased by an average of 52.4% (p = 0.025, Fig. 6B), while that of PD mice was decreased by 40.2% (p = 0.017, Fig. 6B).

We next evaluated immunoreactivity against StAR, HSD3B2, aromatase, HSD17B1, SF-1 and p-CREB in the stromal component of endometriotic lesions (Fig. 6I) since that component has been traditionally regarded as the major site for increased local estrogen production in endometriosis^17,47,52^. We found that StAR and aromatase staining were primarily localized in the cell cytoplasm of stromal cells, but not epithelial cells. HSD3B2 and HSD17B1 stain were seen both in epithelial cells and stromal cells and localized in the cell cytoplasm. SF-1 and p-CREB stain were seen in both epithelial and stromal cells and localized in the cell nucleus. The changes of StAR, HSD3B2, aromatase, HSD17B1, SF-1 and p-CREB in NI group were obviously more as compared with PD group, but obviously less than in PI group.

Consistent with increased lesion weight and also with our *in vitro* findings, we found, by multiple linear regression analysis, significantly higher immunostaining levels of StAR, HSD3B2, aromatase, HSD17B1, SF-1 and p-CREB in the PI group but significantly lower staining levels of all these proteins in the PD group as compared with controls (all p’s < 0.032, R2 ≧ 0.38, Fig. 6C–H). The staining levels of all 6 markers were highly positively correlated (all r’s ≧ 0.52, all p’s < 0.0032). The lesion weight correlated positively with staining levels of all 6 markers involved in estrogen production (all r’s ≧ 0.50, all p’s < 0.0051).

### Antagonism of either TGF-β1 or NF-κB reduces the expression of steroidogenic proteins in endometriotic lesions in mice with induced endometriosis

**Figure 7:**
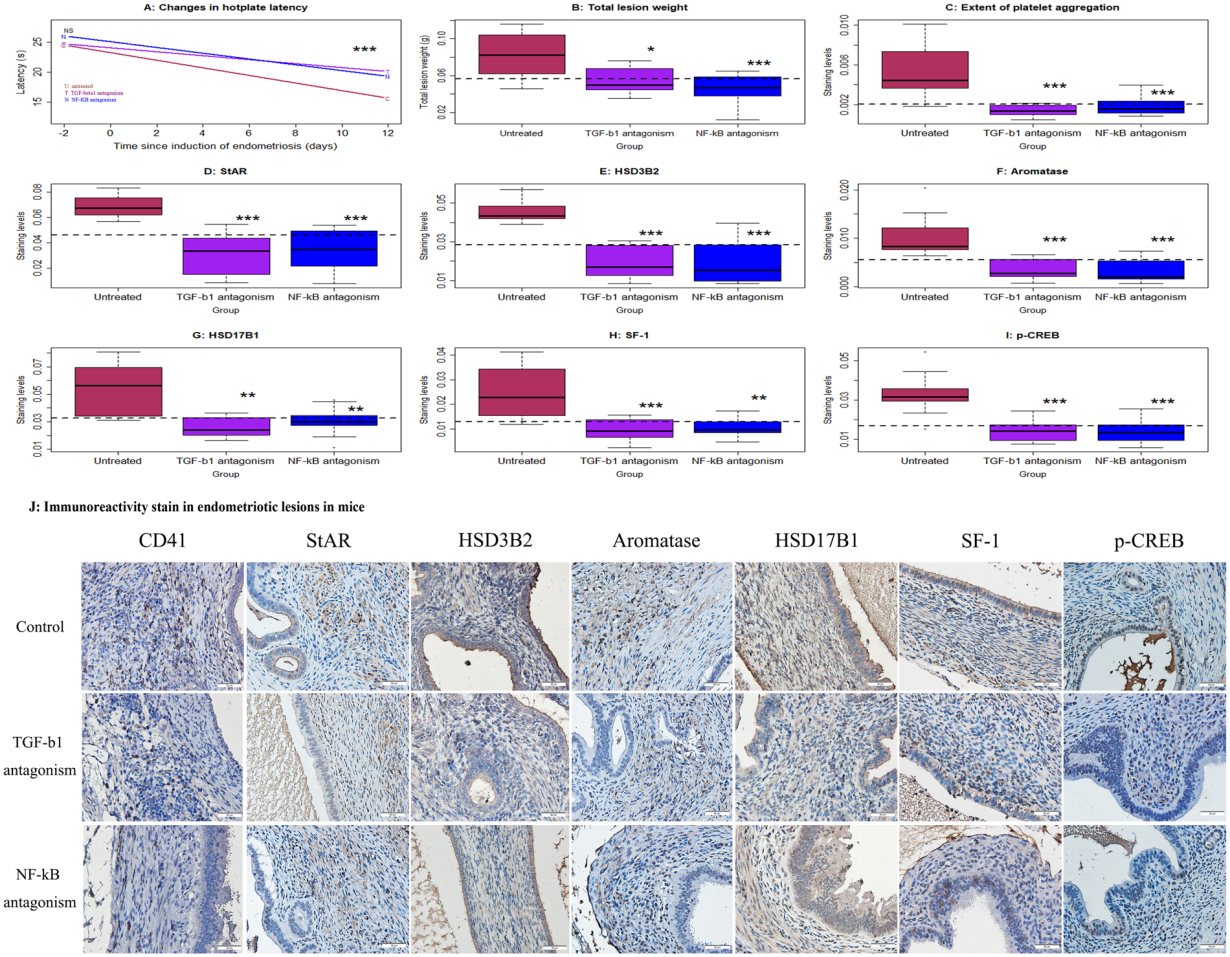
Effect of antagonism of TGF-β1 (by SB431542) or of NF-κB (by JSH-23) on lesional development and lesional expression of steroidogenic proteins in mice with induced endometriosis. (**A**) Change in hotplate latency for mice treatment with vehicle (untreated, or U), antagonism of TGF-β1 (T), and antagonism of NF-κB (N) tested at the time indicated. (**B**) Total lesion weight in the 3 groups of mice. (**C**) Boxplots of the extent of lesional platelet aggregation using CD41 the 3 groups of mice. Boxplots of lesional staining levels in the 3 groups of mice: StAR (**D**), HSD3B2 (**E**), aromatase (**F**), HSD17B1 (**G**), SF-1 (**H**), and p-CREB (**I**). The dotted horizontal line represents the median of all 3 groups. Symbols of statistical significance: *: p < 0.05, **: p < 0.01, ***: p < 0.001. NS: p > 0.05. n = 10 for each group. Except in (**A**), where the comparison was made using Kruskal’s test, all tests were made using Wilcoxon’s test in comparison with the untreated group (**U**). (**J**) Representative immunoreactivity staining of CD41, StAR, HSD3B2, aromatase, HSD17B1, SF-1 and p-CREB in the endometriotic lesions (×400) in Balb/c mice with induced endometriosis which then treatment with vehicle, SB431542 (an antagonist for TGF-β1) and JSH-23 (an antagonist for NF-κB) for two weeks. Scale bar = 50 μm.

We also carried out a mouse experimentation to see whether antagonism of either TGF-β1 or NF-κB could reduce the genes/protein expression in endometriotic lesions in mice with induced endometriosis. Similar to the platelet infusion/depletion experiment as presented above, we found that, while there was no difference in hotplate latency among the 3 groups of mice 2 days prior to the induction of endometriosis, the difference was highly significant 12 days after the induction (p = 0.67 and p = 0.0046, respectively, Fig. 7A). In particular, mice treated with either SB431542 (a TGF-β1 inhibitor) or JSH-23 (an NF-κB inhibitor) had significantly longer (p = 0.008 and p = 0.004, respectively) latency than that treated with vehicle. In addition, the total lesion weight in mice treated with inhibitors of either TGF-β1 or NF-κB was reduced by an average of 65.7% and 34.3%, respectively (p = 0.014 and 0.004, respectively, Fig. 7B) as compared with that in those treated with vehicle.

We also evaluated immunoreactivity against StAR, HSD3B2, aromatase, HSD17B1, SF-1 and p-CREB in the stromal component of endometriotic lesions, as well as the extent of platelet aggregation using CD41 as a marker (Fig. 7J), as platelets aggregated mostly in lesional stromal component^23^. We can see that, compared with mice treated with vehicle, mice treated with either SB431542 or JSH-23 had significantly reduced platelet aggregation, and immunostaining levels of StAR, HSD3B2, aromatase, HSD17B1, SF-1 and p-CREB in the stromal component of endometriotic lesions (all p’s < 0.007, Fig. 7C–I).

As expected, the extent of platelet aggregation correlated positively with staining levels of all 6 markers involved in estrogen production (all r’s ≧ 0.53, all p’s < 0.0027), confirming the roles of platelets in estrogen biosynthesis in endometriotic lesions. In addition, the lesion weight correlated positively with staining levels of all markers involved in estrogen production (all r’s ≧ 0.45, all p’s < 0.012) except SF-1 (r = 0.28, p = 0.14), which is upstream of aromatase.

## Discussion

In this study, we have shown through *in vitro* and *in vivo* studies that activated platelets increase the E_2_ production in endometriotic stromal cells through upregulation of StAR, HSD3B2, aromatase, and HSD17B1. In addition, platelets activate these genes through the activation of NF-κB and/or TGF-β1, and antagonism of either signaling pathway can abolish the induction of the 4 genes and thus the estrogen production. Platelets also induce HIF-1α, SF-1 and p-CREB, suggesting that the platelet-induced estrogen overproduction can be achieved in multiple pathways. Remarkably, the product of the fold increase of the 4 proteins after platelet stimulation is nearly equal to the fold increase in E_2_ production in endometriotic stromal cells (4.1 vs. 4.5), suggesting that, first, the activated platelets are indeed responsible for the increased E_2_ production through activation of these 4 genes. Second, it suggests that other genes, such as CYP11A1 and CYP17A1 (Fig. 1), that are involved in the synthesis of E_2_ may also be involved but we were not able to detect them simply because of either the fold increase is small or there is lack of adequate statistical power due to small sample sizes used in our study.

**Figure 8:**
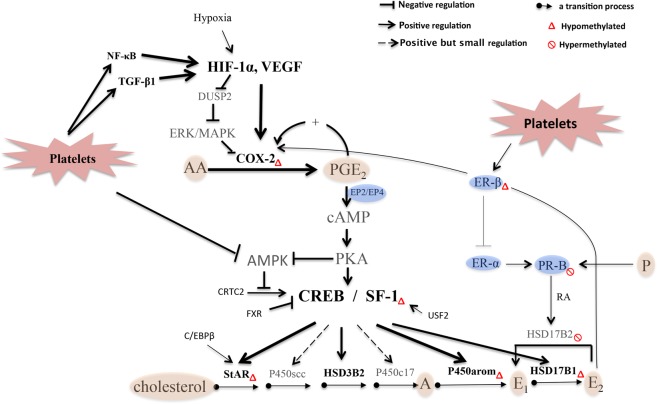
A schematic diagram that depicts platelet-induced estrogen biosynthesis in endometriosis. Activated platelets induce the activation of the NF-κB signaling pathway and TGF-β1/Smad3 signaling pathway in endometriotic lesions^21^. The two signaling pathways regulate several genes coding for hypoxia and inflammatory molecules, such as HIF-1α, COX-2 and VEGF^99–101^, which are upregulated after platelets treatment in HESCs. Activated platelets induce hypoxia in endometriotic lesions as shown by elevated expression of HIF-1α and VEGF. Increased HIF-1α accumulation results in DUSP2 down-regulation, which results in prolonged activation of ERK and p38 MAPK and increased COX-2 expression in endometriotic stromal cells^49^, as well as increased ERβ expression^66^ which can also be induced by activated platelets. The COX-2 overexpression leads to increased production of PGE_2_ in endometriosis, further favor E_2_ synthesis in HESCs. High levels of local E_2_ (through ERβ) and PGE_2_ in turn may further amplify COX-2 expression^83^, which results in overexpression of steroidogenic genes, and continuous local production of E_2_ and PGE_2_ in endometriotic tissue. PGE_2_ increases intracellular cAMP levels via the receptors EP2 or EP4^102^. Elevated cAMP upregulates SF-1 and enhances its binding to the promoter of steroidogenic genes^15^, as well as PKA activation, resulting in the phosphorylation of CREB which facilitates its binding to a cAMP-response element (CRE) on the promoter region of the StAR, aromatase or some other steroidogenic genes^45,103^, and acts as an initiator to unfold the DNA-histone binding and then provides space for C/EBP binding to the promoter of these steroidogenic genes. PGE_2_-induced StAR promoter activity appears to be regulated by CREB and C/EBPβ in a cooperative manner in endometriotic stromal cells^16^. PGE_2_ negatively regulates AMPK via the PKA signaling pathway^104^, and CREB and related proteins are also the direct downstream targets for AMPK^105^. AMPK is crucial for the activation of CREB via phosphorylation and there is a negative regulatory role of AMPK in the expression of CREB^106^. CRTC2 is translocated into the nucleus, where it forms a transcription complex with CREB^107^. AMPK can directly phosphorylate CRTC family members. In the absence of AMPK activity, CRTC2 is dephosphorylated and translocated to the nucleus and associates with CREB, and then increases target gene expression^108^. Activated platelets also inhibit AMPK activation via downregulation the phosphorylation level of AMPK in HESCs (Qi *et al*., unpublished data), which can further activate the CREB binding to steroidogenic genes in HESCs. In addition, it reported that USF2 binds to the SF-1 promoter, and stimulates the expression of SF-1 and its target genes^109^, and recent research has shown that FXR competes with CREB in binding the promoter region of CYP19A1, which results in the downregulation of aromatase expression^110^. Progesterone induces the expression of epithelial HSD17B2 by activating stromal PR-B, which can mediate the formation of retinoid acid to bind to the promoter and thus activate HSD17B2. In endometriotic tissues, the decreased PR-B expression in stromal cells disrupts the paracrine action of progesterone. ERβ suppresses ERα and PR-B, leading to progesterone resistance and deficient inactivation of E_2_ in endometriotic lesions. Abbreviations used: AA: arachidonic acid, PGE_2_: prostaglandin E_2_, RA: retinoic acid, COX-2: cyclooxygenase-2, A: androstenedione, E_1_: estrone, E_2_: estradiol, P: progesterone, SF-1: steroidogenic factor-1, CREB: cAMP-response element-binding protein, StAR: steroidogenic acute regulatory, P450scc: cytochrome P450 side-chain cleavage, HSD3B2: 3-hydroxysteroid dehydrogenase type 2, P450c17: cytochrome P450 17α-hydroxylase/17, 20-lyase, HSD17B1: 17beta-hydroxysteroid dehydrogenase type 1, HSD17B 2: 17beta-hydroxysteroid dehydrogenase type 2. ER-α: Estrogen receptor-α; ER-β: Estrogen receptor-β. PR-B: progesterone receptor B, ERK: extracellular signal-regulated kinase, AMPK: AMP-Activated Protein Kinase, CRTC2: CREB-regulated transcription coactivator 2, C/EBPβ: CCAAT/enhancer binding protein β, USF2: Upstream stimulatory factor 2, FXR: Farnesoid X Receptor.

While endometriosis has been traditionally viewed as an estrogen-dependent disease as well as an inflammatory condition, the notion that endometriotic lesions are wounds undergoing ReTIAR is only appreciated very recently^21,22,53^ despite the fact that endometriotic lesions exhibit dynamic changes in immune cell populations that are uncannily similar to that of a healing wound^54^. In particular, the roles of platelets in the development of endometriosis has been demonstrated only recently^23^, despite the disease is characterized conspicuously by cyclic bleeding^55^. Indeed, estrogen is very important to wound healing^27,28^ and so is ERβ^37,38^ in wound healing, another similarity that cannot be dismissed as just a pure coincidence. In essence, this study shows that, as wounds undergoing ReTIAR, platelets also participate in the increased local production of estrogens through various ways. We summarize the roles of platelets in estrogen biosynthesis in endometriotic lesions in Fig. 8.

Our results are consistent with our previous report that platelets induce the TGF-β1/Smad3 signaling pathway in endometriotic cells^21^. The result of 4.5 fold increase in estrogen production in endometriotic stromal cells stimulated with activated platelets vs. those unstimulated is remarkably close to the reported fold increase found between testosterone-added endometriotic and endometrial stromal cells (4.1 for follicular phase and 4.5 for luteal phase)^41^. Previous studies have reported that the TGF-β1/Smad3 signaling pathway is involved in the transcriptional regulation of aromatase in several cell types^56–58^, and particularly in endometriotic stromal cells^59^. Therefore, our finding is also consistent with these reports. Our results are also consistent with the report that activated platelets activate NF-κB in cancer cells^60^. The resultant hypercoagulability in women with endometriosis^61,62^ may be responsible for increased risk of preeclampsia^63,64^ due to inflammasome activation in trophoblasts induced by maternal extracellular vesicles and platelets^65^.

Our results are consistent with the finding that activation of HIF-1α and thus hypoxia can lead to increased estrogen production through depressing DUSP2 expression and thus COX-2 overexpression in endometriotic stromal cells^49^, as well as increased ERβ expression^66^. What is different, however, is that instead of invoking the assumption of endometrial fragments regurgitated into the peritoneal cavity that are deprived of oxygen^66^, our study made a simpler and more natural assumption that platelets that are activated due to ReTIAR and platelet activating factors secreted by endometriotic stromal cells^67^.

While endometriosis has always been defined as “the presence of endometrial glands and stroma outside of the normal location”^68^, the lesion does not develop, exist or even take its root totally on its own. Granted, while almost all past research focus has fixated on endometriotic epithelial and stromal cells nearly exclusively, there are many other cells in its microenvironment. Platelets^21,23^, neutrophils^69^, macrophages^19,24^, and recently nerve fibers^25,26^ all have been identified to be accessories to aid and abet the lesional development. Indeed, these cells are known to be irreplaceable partners in a successful wound healing^70^. The absence or over-abundance of any of these cell types would derail the normal healing process, leading healing astray and causing pathological healing such as fibrosis^70^.

Our findings give more credence to the notion that endometriotic lesions are wounds undergoing ReTIAR. Indeed, peripheral tissues produce estrogens^71^, and estrogens play a vital role in wound healing^27,28^. While normal endometrial stromal cells only produce negligible levels of estrogens^41^ likely due to a silenced aromatase because of hypermethylation^41,42^, endometriotic stromal cells, in contrast, do produce a much higher level of estrogens, due to demethylated aromatase^42^, and overexpression of StAR, HSD3B2 and HSD17B1^12,43,45,47^. Moreover, it has been reported that estrogen promotes wound healing mainly through ERβ, not ERα^38^. Concomitant with declining wound healing ability, epidermal ERβ expression declines with age in both sexes^72^. And this may explain as why ERβ, but not ERα, is overexpressed in endometriosis^35,36^. Incidentally or not, we have shown previously that ERβ expression can also be upregulated by activated platelets^73^.

Our finding that platelets play an important role in estrogen production in endometriotic stromal cells has several important clinical implications. Currently, all existing drugs for treating endometriosis are hormonal^74^, with the aim of reducing estrogen production through induction of state of pseudo-pregnancy, pseudo-menopause, or chronic anovulation, thus creating an acyclic, hypoestrogenic environment^75^. In essence, this therapeutic approach is based on the idea of cutting off the fuel supply to lesion growth.

Along this line, there is a hypothesis of treatment window, that pharmacologically reduces the estrogen level to a certain level so that the growth of ectopic endometrium is curtailed or contained but the level is not too low to cause any detrimental side-effects due to hypoestrogenism^76^. The recent approval of the GnRH antagonists for treating endometriosis is more or less based on this notion. However, this notion only works if the following two implicit assumptions hold true: 1) the inter-individual variation in the treatment window is small, and 2) the inter-individual variation in response to drug treatment also is small. If either assumption is violated, this notion is unlikely to be therapeutically feasible.

However, the findings from this study justify the use of non-hormonal drugs for therapeutic purpose. They essentially show that, just by suppression of platelet activation either through anti-platelet treatment or neutralization of NF-κB or of TGF-β1, the lesional production of estrogen can be substantially curtailed or even minimized to achieve the desired therapeutic effect without messing up the hormonal system.

This study has several strengths. First, through highlighting the importance of platelets in lesional development, and, in this case, estrogen production, this study provides yet another piece of evidence that endometriotic lesions are indeed wounds undergoing ReTIAR. Second, through the use of both *in vitro* and *in vivo* studies, we have provided convincing evidence that platelets are an unindicted culprit responsible for increased local production of estrogen, and that this is mainly through the induction of NF-κB and TGF-β1 signaling pathways. Third, our study provides evidence that activated platelets can directly induce a hypoxia state in endometriotic lesions, which subsequently cause further phenotypic changes^39^. Lastly, this study further underscores the importance of lesional microenvironment in shaping its fate.

Our study also has some limitations. First, we did not evaluate all genes that are possibly involved in estrogen biosynthesis in endometriotic stromal cells, and focused mainly on StAR, aromatase, HSD3B2 and HSD17B1, in which the product of their fold increase seemingly accounted for most of the variation in estrogen production in endometriosis. In addition, we also evaluated their upstream genes, such as HIF-1α, SF-1 and CREB. However, there are other genes/enzymes that have been reported to be involved in the increased estrogen production in endometriosis, such as AMPK^77^. While this may be viewed as a deficiency, we note that any study, by necessity, needs to be limited by its aim and scope, and simply cannot evaluate all possible genes/enzymes involved in a complex process such as this in one single study. Hopefully, our study can pique the interest in the ultimate cause for estrogen overproduction in lesions in future studies.

Second, while we have identified NF-κB and TGF-β1 signaling pathways that are involved in lesional estrogen overproduction, we did not fully elucidate the exact molecular mechanisms in more detail, nor did we demonstrate just exactly how platelets activate NF-κB and/or TGF-β1. Again, this should await further investigation, and it is possible that platelet-activating factor (PAF) may activate NF-κB^78^. It is also possible that NF-κB is activated by platelets in a direct signaling manner as in cancer cells^60^. Future studies are warranted to clarify this mechanism.

Lastly, we only employed ovarian endometrioma tissue samples in the *in vitro* study. Given the known heterogeneity and complexity among different subtypes of endometriosis^79^, this raises the question whether the conclusions reached from this study apply to all subtypes of endometriosis. As we showed previously, however, the vast heterogeneity of endometriosis appears to be determined mostly by the lesional microenvironments^80^. Namely, regardless of subtypes, all endometriotic lesions appear to undergo identical molecular processes such as epithelial-mesenchymal transition (EMT), fibroblast-to-myofibroblast transdifferentiation (FMT), smooth muscle metaplasia (SMM), and fibrogenesis^25,26,53^, even in adenomyotic lesions^81,82^. Moreoever, increased local production of estrogen is common to all subtypes of endometriosis^83^ and even to adenomyosis^84^. Therefore, in this context, the conclusions reached by this study are likely to be true to all subtypes of endometriosis.

In summary, we have demonstrated that activated platelets increase the E_2_ production in endometriotic stromal cells through upregulation of StAR, HSD3B2, aromatase, and HSD17B1 via the activation of NF-κB and/or TGF-β1. In addition, the increased lesional estrogen production by activated platelets provides a compelling piece of evidence that endometriotic lesions are indeed wounds undergoing ReTIAR. Our findings strongly indicate that non-hormonal therapeutics for endometriosis is theoretically viable, with anti-platelet therapy being one promising avenue.

## Materials and Methods

### Patients and specimens

This study strictly adhered to the ethical principles outlined by the Helsinki Declaration and was approved by the institutional ethics review board of Shanghai OB/GYN Hospital, Fudan University. After informed consent, endometriotic tissue samples were obtained from 15 premenopausal patients (mean age = 32.2 ± 5.2 years) with surgically diagnosed and histologically confirmed ovarian endometriomas, who were admitted to the Shanghai OB/GYN Hospital, Fudan University, from May 2015 to March 2016. The harvested endometriotic tissue samples were used for deriving primary endometriotic stromal cells, which were then used for ELISA analysis, real-time PCR analysis and Western blotting analysis.

### Cell culture and reagents

Endometriosis-derived primary human endometriotic stromal cells (HESCs) were isolated and cultured as reported previously^23,73^. Briefly, the endometriotic tissues were minced into small pieces of about 1 mm³ in size, after washing with DMEM/F-12 medium (Dulbecco’s modified Eagle’s medium/Ham’s F-12 medium, Hyclon, Logan, UT, USA) supplemented with 100 IU/mL penicillin and 100 mg/ml streptomycin (Invitrogen, Paisley, UK). After the enzymatic digestion of minced tissues with 0.2% collagenase II (Sigma, St Louis, MO, USA) in a shaking bed for about 1.5 hours at 37 °C, they were separated by filtration through a 149 μm and then a 37 μm (all pore sizes) nylon mesh. The stromal cells remaining in the filtrate were collected by centrifugation, re-suspended in DMEM/F12 (reconstituted with 10% charcoal-stripped fetal bovine serum (FBS, Gibco Laboratories, Invitrogen, Auckland, New Zealand) and 1% antibiotics), and seeded into 25 cm² cell culture flasks and incubated at 37 °C in humidified atmosphere of 5% CO_2_ in air. The purity and homogeneity of the stromal cell preparation (≥98%) were verified by immunocytochemistry with an antibody against vimentin (Abcam, Cambridge, UK), a specific marker of stromal cells, and an antibody against cytokeratin 7 (CK7) (CK-7, Abcam), a specific marker of epithelial cells, and, finally, an antibody against follicle-stimulating hormone receptor (FSHR, Abcam), a specific marker for granulosa cells (used to rule out the possibility of contamination with ovarian granulosa cells). After 3–4 passages, the vimentin staining was positive, while CK7 staining and FSHR staining were negative. Then, the isolated cells were seeded into cell culture plates for further analyses. The human endometrial stromal cell line (ESCL), established by Dr. Krikun and her colleagues^85^, was kindly provided by Dr. Asgi Fazleabas, and cultured in DMEM/F-12 medium (Hyclone) supplemented with 5% FBS and 1% antibiotics.

HESCs were added to the serum-free DMEM/F-12 medium at about 80% confluence and were starved for 24 hours before proceeding experiments. To see the effect platelets may have on endometriotic stromal cells, HESCs were incubated with 3.5 mL of different treatment media for 48 hours as follows: PBS group (standard medium + PBS, i.e. phenol red free DMEM/F-12 medium (Gibco Laboratories, Life Technologies, Grand island, NY, USA) with 10% charcoal-stripped FBS), platelets group (standard medium containing 10^7^ human platelets), activated platelets group (standard medium containing 10^7^ human platelets and human thrombin (1.49 NIH U (Sigma, St. Louis, MO, USA)), and thrombin group (standard medium containing human thrombin 1.49 NIH U). To examine the potential molecular mechanisms that underlie activated platelets-induced estrogen production, HESCs were pretreated with 30 μM JSH-23 compound (an NF-κB inhibitor, Santa Cruz, CA, USA) for 6 hours or with 1 μM A83-01 compound (a TGF-β receptor inhibitor, Santa Cruz) for 2 hours, and then incubated with activated platelets plus JSH-23 or A83-01 for 48 hours.

To ensure that the real-time PCR and Western blot analysis results refer to HESCs but not platelets, we washed the HESCs that co-cultured with platelets with sterile PBS three times to remove the platelets as reported previously^86^. The supernatant of HESC and ESCL, after treatment with PBS, platelets, activated platelets (by thrombin stimulation) and thrombin alone for 48 hours, was collected for ELISA analysis.

### Preparation of platelets

Platelets, donated by healthy male volunteers who denied taking any medications for at least two weeks prior to donation after informed consent, were isolated from the whole blood samples by centrifugation at room temperature as reported previously^23,73^. The blood was first centrifuged at 150 g for 10 min, the supernatant platelet rich plasma (PRP) was then centrifuged at 300 g for 5 min, and the supernatant PRP was harvested again and then centrifuged at 1000 g for 14 min. Finally, the depositing platelets were suspended in DMEM/F12 culture medium with 10% FBS for cell culture experiments, yielding ~2 × 10^8^ platelets from 20 mL of peripheral blood samples from each volunteer.

### ELISA

To determine the concentration of E_2_ in the supernatants, the Human 17β-estradiol ELISA Kit (Abcam) was used according to the manufacturer’s protocol. Briefly, purified human E_2_ was used to coat the microtiter plate wells to form solid-phase antibody. The aqueous samples were added to individual wells. E_2_ present in the aqueous combined with horseradish peroxidase (HRP)-labeled antibody to form antibody-antigen-enzyme-antibody complex. After washing thoroughly with prepared washing solution, tetramethylbenzidine (TMB) substrate solution was added, turning the HRP-enzyme-catalyzed TMB substrate blue. The reaction was terminated by the addition of a sulphuric acid solution, and the color change was measured spectrophotometrically at a wavelength of 450 nm. The concentration of E_2_ in the samples was then determined by comparing the optical density (OD) of the samples to the standard curve. We plotted the OD of the standards against the standard concentration on graph, and draw the curve through these points to construct the standard curve (R^2^ = 0.99). The concentrations for unknown samples were determined from the standard curve. For each sample, the concentration of E_2_ was measured twice, and the average was used as the concentration of E_2_ in that sample.

### RNA isolation and real-time PCR

After co-culture with different media for 48 hours, the total RNA was isolated from HESCs using Trizol reagent (Invitrogen, UK), and cDNA synthesis was performed using the reverse transcription kit (Takara, Otsu, Japan). We measured the expression level of the estrogen production related genes, StAR, HSD3B2, CYP19A1 and HSD17B1 and downstream molecules of NF-κB, HIF-1α, COX-2, and VEGF by RT-PCR using SYBR Premix Ex Taq (Takara, Otsu, Japan). The oligonucleotide primer sequences (synthesized by Biotnt Corporation, Shanghai, China) used this study are listed in Table 1. Melting curves of the products were obtained after cycling by a stepwise increase of temperature from 55 °C to 95 °C. At the end of 40 cycles, the reaction products were separated electrophoretically on an agarose gel and stained with ethidium bromide for visual confirmation of the PCR products. The expression values were normalized to the geometric mean of GAPDH measurements and the quantification of mRNA abundance was made using the method as described in^87^.

**Table 1:** List of primer sequences for RT-PCR used in this study.

Gene name	Forward sequence	Reverse sequence
StAR	5′ TGT ACC CAC CTA AAA CCA TC 3′	5′ CCC ATA AAG CAA GAC TTC TC 3′
CYP11A1	5′ TCC AGA AGT ATG GCC CGA TT 3′	5′ CAT CTT CAG GGT CGA TGA CAT AAA 3′
HSD3B2	5′ GCC ACA CAG TCA CAT TAT CA 3′	5′ ACT CCA CGG TTT TCT GCT T 3′
CYP17A1	5′ TCT CTG GGC GGC CTC AA 3′	5′ AGG CGA TAC CCT TAC GGT TGT 3′
CYP19A1	5′ TAA CAC GCT CTT CTT GAG GA 3′	5′ AGA TGT CTG GTT TGA TGA GG 3′
HSD17B1	5′ GAT CCT ACA ACC CAC AAT CA 3′	5′ AAA ACA GCC TTT CGT CTC TC 3′
COX-2	5′ GCT CAA ACA TGA TGT TTG CAT TC 3′	5′ GCT GGC CCT CGC TTA TGA 3′
VEGF	5′ CGA AAC CAT GAA CTT TCT GC 3′	5′ CCT CAG TGG GCA CAC ACT CC 3′
HIF-1α	5′ GTC GAC ACA GCC TGG ATA TGA A 3′	5′ CAT ATC ATG ATG AGT TTT GGT CAG ATG 3′
GAPDH	5′ TGC ACC ACC AAC TGC TTA G 3′	5′ GAT GCA GGG ATG ATG TTC 3′

### Western blot analysis

For total protein extraction, HESCs were scraped and extracted in commercialized RIPA buffer (Thermo, Waltham, MA, USA). The protein concentration, after co-cultured with different media for 48 hours, was determined using BCA protein quantitative analysis kit (P0010S, Beyotime, Shanghai, China). All proteins mixed with SDS-PAGE loading buffer (P0015, Beyotime) were heated for 10 min at 95 °C for denaturalization. The protein samples were loaded on a 10% SDS-PAGE, and electro-blotted onto PVDF membranes (Bio-Rad, Hercules, CA, USA). The membranes were blocked in Western blocking buffer (P0023B, Beyotime) for 1 hour at room temperature and subsequently incubated at 4 °C overnight with the primary antibodies as listed in Table 2. After the membranes were incubated with HRP-labeled secondary antibodies for 1 hour at room temperature, the signal was detected using ECL (Pierce, Thermo Scientific, Rockford, IL, USA) on Image Quant LAS 4000 mini. The protein expression levels were quantified by Quantity One software (Bio-Rad).

**Table 2:** List of primary antibodies for western blot analysis used in this study.

Antibody name	Dilutions	Vendor name	Cat. No.
StAR	1:1000	Abcam, Cambridge, UK	Ab203193
HSD3B2	1:1000	GeneTex, Alton, CA, USA	GTX102813
Aromatase	1:1000	Abcam, Cambridge, UK	Ab18995
HSD17B1	1:1000	Thermo, Waltham, MA, USA	PA5–42058
SF-1	1:1000	Abcam, Cambridge, UK	Ab65815
p-CREB	1:1000	Abcam, Cambridge, UK	Ab32096
CREB	1:1000	Abcam, Cambridge, UK	Ab32515
COX-2	1:1000	Abcam, Cambridge, UK	Ab6665
VEGF	1:1000	Abcam, Cambridge, UK	Ab46154
HIF-1α	1:1000	Abcam, Cambridge, UK	Ab16066
p65	1:1000	Cell Signaling Technology, MA, Boston, USA	Ab10437
p-p65	1:1000	Cell Signaling Technology, MA, Boston, USA	Ab193238
GAPDH	1:1000	Cell Signaling Technology, Boston, MA, USA	8884

In Western blot analysis using different primary antibodies, we cropped the polyvinyl difluoride (PVDF) membranes since antibodies against different proteins may require slightly different treatment. However, when only a single primary antibody was used, no cropping was performed.

### Mouse experiments

A total of 90 virgin female Balb/C mice, ~6 weeks old and about 16–18 g in bodyweight, were purchased from SLAC Experimental Animal Company (Shanghai, China) and used in two experiments. All mice were maintained under controlled conditions with a light/dark cycle of 12/12 hours and had access to food and water adlibitum. All our experiments were performed following the guidelines of the National Research Council’s Guide for the Care and Use of Laboratory Animals^88^ and approved by the institutional experimental animals review board of Shanghai OB/GYN Hospital, Fudan University.

We used an established mouse model of endometriosis^89^ by intraperitoneal (i.p.) injection of endometrial fragments as our previous studies^23,90^. Briefly, after one week of acclimatization, 7-week-old donor mice were intramuscular injected with 3 μg/mouse estradiol benzoate (Animal Medicine Factory, Hangzhou, China) to stimulate the growth of endometrium. One week later they were sacrificed and their uteri were harvested. The uterine tissues were seeded in a Petri dish containing warm sterile saline, and split longitudinally with a pair of scissors.

Two uterine horns from each mouse were minced with scissors, ensuring that the maximal diameter of the fragment was consistently smaller than 1 mm. The fragments were then injected i.p. to recipient mice. To minimize any individual variation, the endometrial tissue fragments from two donor mice were mixed together and then divided into three or four parts, each injected i.p. to one mouse each from one of the three or four groups, depending on the experiment design.

The first experiment evaluated the effect of platelet infusion and platelet depletion on the immunoreactivity against proteins involved in estrogen biosynthesis in endometriotic lesions. The second experiment, in contrast, evaluated the effect of NF-κB or TGF-β1 antagonism on the immunoreactivity against proteins involved in estrogen biosynthesis in lesions.

In the first experiment, 45 mice were used. Among the 45 mice, 15 were randomly selected as donors, and the remaining 30 were recipients who received endometrial tissues from donor mice. Ninety male mice of similar age and the same strain were used mainly for harvesting platelets. Briefly, two days (Day-2) before the induction of endometriosis (Day 0), all recipient mice were weighed and subjected to a baseline hotplate test. The mice were randomly divided into three groups of 10: PI, PD and NI. In the PI group, platelets (4 × 10^7^/mouse) were given intravenously on Days −2, 3 and 8. In PD and NI groups, rat anti-mouse GPIba polyclonal IgG or rat anti-mouse isotope-matched non-immune polyclonal IgG respectively (both from Emfret Analytics, 1.5 mg/g bodyweight) were given intravenously on Days −2, 3 and 8. The concentration was determined following the manufacturer’s instructions and our previous study^23^.

JSH-23 and SB-431542 can suppress NF-κB and TGF-β1/Smad3 signaling pathways respectively and thus might inhibit platelet aggregation in endometriosis^91,92^. In the second experiment, 30 mice were randomly divided into three groups of equal size: control group (CT), NF-κB antagonism group (NA), and TGF-β1 antagonism group (TA). Mice in the NA, TA, and CT groups received i.p. injection of SB-431542 (10 mg/kg, MCE, Shanghai, China)^93^, JSH-23 (20 mg/kg, MCE)^94^, and equal volume of vehicle very other day (i.e. days −2, 0, 2, 4, ..., 10 and 12), starting from 2 days before the induction of endometriosis (day 0, the day when induction was performed)^95^. Two days after the first injection, endometriosis was induced as described above for all mice. Two days before and 12 days after induction, all mice were weighed and subjected to hotplate test. All mice were sacrificed by cervical dislocation and their lesions were excised, weighed, and processed for further evaluation or immunohistochemistry.

### The hotplate test and lesion measurement

To evaluate endometriosis-associated hyperalgesia in mice, hotplate test was administrated to all mice using a Hot Plate Analgesia Meter (Model BME-480, Institute of Biomedical Engineering, Chinese Academy of Medical Sciences, Tianjin, China) as reported previously^23^. The extent of endometriosis was measured by measuring the dry weight of all lesions excised from mice as described in^96^. The endometriotic lesions were excised and carefully weighed and then fixed with 10% formalin (w/v) for IHC and histochemistry analyses.

### Immunohistochemistry (IHC) staining

Serial 4 μm sections were obtained from each paraffin-embedded tissue block, with the first resultant slide was stained with hematoxylin and eosin (H&E) to confirm pathologic diagnosis, and the subsequent slides for IHC analysis for StAR, HSD3B2, aromatase, HSD17B1, SF-1 and p-CREB. Routine deparaffinization and rehydration procedures were performed. The optimal concentrations of these primary antibodies were shown in Table 3. The slides were heated at 98 °C in a citrate buffer (pH 6.0) for a total of 30 min for antigen retrieval, cooled naturally to the room temperature, and were washed 3 times in PBS 0.5% goat serum for blocking nonspecific binding sites. Sections were then incubated overnight with the primary antibody at 4 °C. After slides were rinsed, the biotinylated secondary antibody, Supervision TM Universal (Anti-Mouse/Rabbit) Detection Reagent (HRP) (Shanghai GeneTech Company, Shanghai, China), was incubated for 30 min at room temperature. The bound antibody complexes were stained for 3–5 min or until appropriate for microscopic examination with diaminobenzidine and then counterstained with hematoxylin and mounted. Immunostaining results were evaluated using a semi-quantitative scoring system, as reported previously^97^. Briefly, images were obtained with the microscope (Olympus BX51, Olympus, Tokyo, Japan) fitted with a digital camera (Olympus DP70, Olympus) and exported as a TIFF-format digital file, and then used IPP (Image-Pro Plus, version 6.0, Media Cybernetics, Inc., Bethesda, MD, USA) to count the number or the intensity of positive stain, blind to which group the slide belonged to. A series of 4–5 images randomly selected from several sections per tissue sample were taken for each immunostaining parameter to obtain a mean value. The positive and negative controls are shown in Supplementary Fig. S2.

**Table 3:** List of primary antibodies for immunohistochemistry analysis in this study.

Antibody name	Dilutions	Vendor name	Cat. No.
StAR	1:100	Abcam, Cambridge, UK	Ab203193
HSD3B2	1:100	GeneTex, Alton, CA, USA	GTX102813
Aromatase	1:100	Abcam, Cambridge, UK	Ab18995
HSD17B1	1:100	Thermo, Waltham, MA, USA	PA5–42058
SF-1	1:100	Abcam, Cambridge, UK	Ab65815
p-CREB	1:200	Abcam, Cambridge, UK	Ab32096
CD41	1:100	Abcam, Cambridge, UK	Ab33661

### Statistical analysis

To compare the treatment effect in *in vitro* experimentations, paired Wilcoxon’s test was used. The comparison of distributions of continuous variables between the two groups was made using Wilcoxon’s test. Pearson’s correlation was used when evaluating correlations between two variables. To see whether factor is associated with the immunostaining levels of a given marker when more than one factor is involved (such as platelet infusion and depletion), multiple linear regression analysis was used. A p-value of less than 0.05 was considered statistically significant. All calculations were carried out using the software R (version 3.5.1)^98^ or SPSS (version 16.0).

## Supplementary information

Supplementary information.
