# Supplementary material for: Platelets induce increased estrogen production through NF-κB and TGF-β1 signaling pathways in endometriotic stromal cells
Source: Sci Rep. 2020 Jan 28;10:1281. doi: 10.1038/s41598-020-57997-6 (PMC6987096; doi:10.1038/s41598-020-57997-6)

**Supplementary Information**

**Platelets induce increased estrogen production through NF-κB and TGF-β1 signaling pathways in endometriotic stromal cells**

**Qiuming Qi, Xishi Liu, Qi Zhang, Sun-Wei Guo**


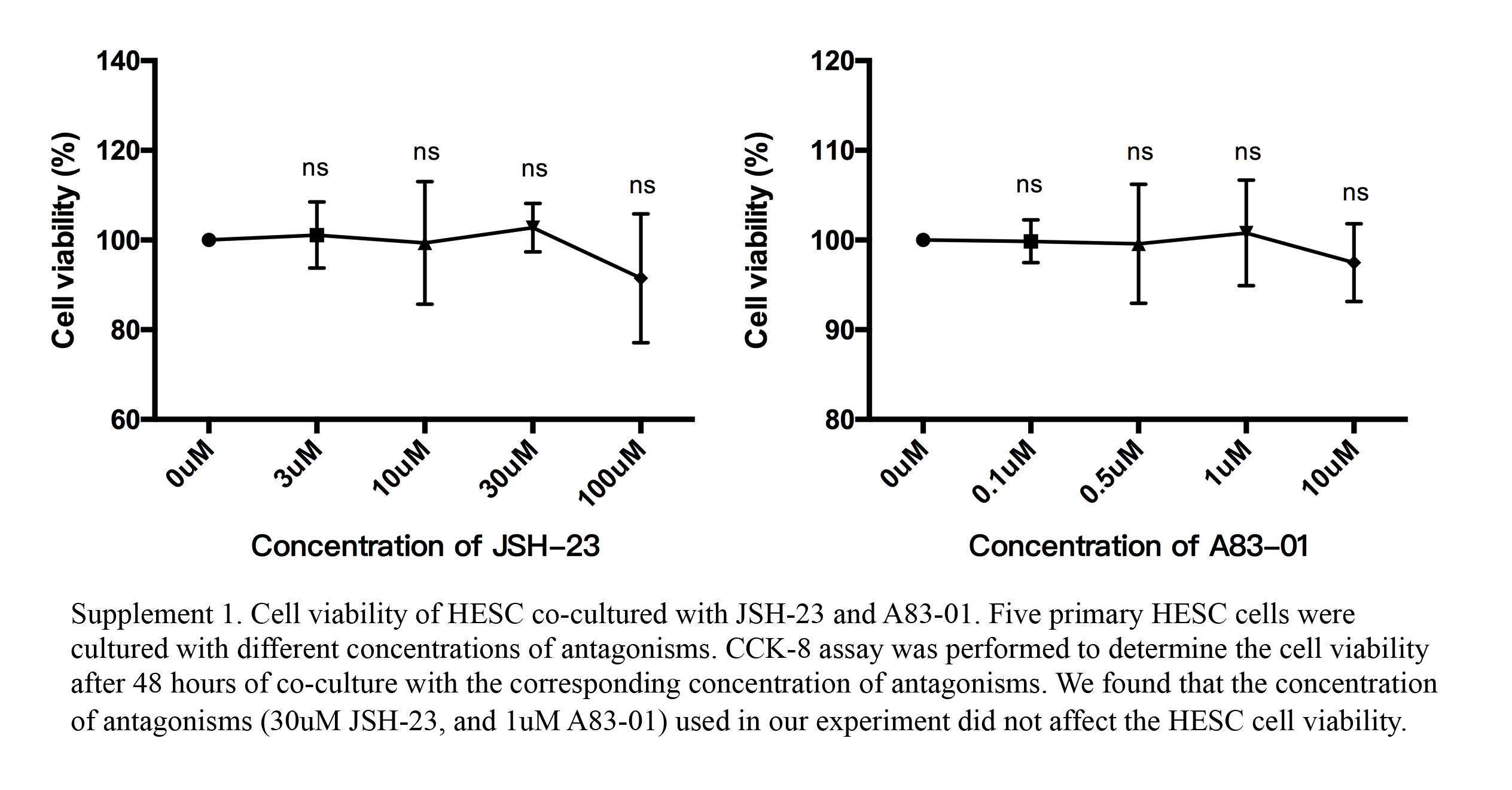


**Supplementary Figure S1**. Cell viability of HESC co-cultured with JSH-23 and A83-01. Primary HESC cells derived from 5 patients were cultured with different concentrations of antagonist (JSH-23 or A83-01). CCK-8 assay was performed to determine the cell viability after 48 hours of co-culture with the corresponding concentration of the designated antagonist. The concentration of antagonisms (30 μM JSH-23, and 1 μM A83-01) used in our experiment did not affect the HESC cell viability. “ns” means no statistical difference (i.e. p>0.05) as compared with the control group (treated with no i.e. 0 μM) In each dosage group, number of replication was 5. Paired Wilcoxon’s test was used.


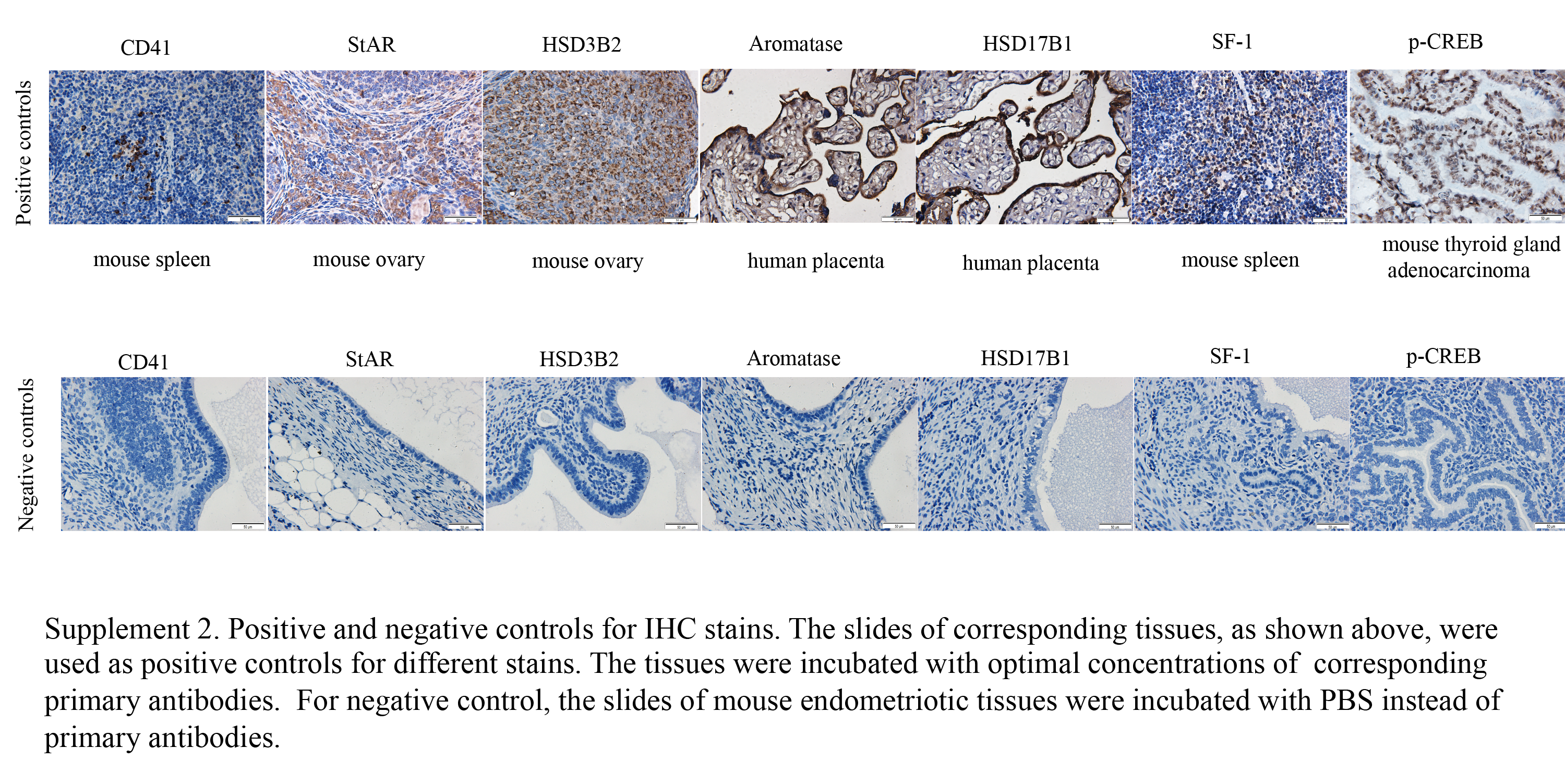


**Supplementary Figure S2.** Positive and negative controls for immunohistochemistry analysis. For positive control, mouse spleen tissue was used for CD41 and SF-1, mouse ovary was used for StAR and HSD3B2, human placenta was used for aromatase and HSD17B1, and mouse thyroid gland adenocarcinoma was used for p-CREB. The tissues were incubated with optimal concentrations of corresponding primary antibodies. For negative control, the slides of mouse endometriotic tissues were incubated with corresponding serum instead of primary antibodies. The scale bar=50μm.

**Supplementary materials for Western blots**

***A lower exposure version of GAPDH blots in Figures 2 and 5.***

GAPDH-Figure 2D↓


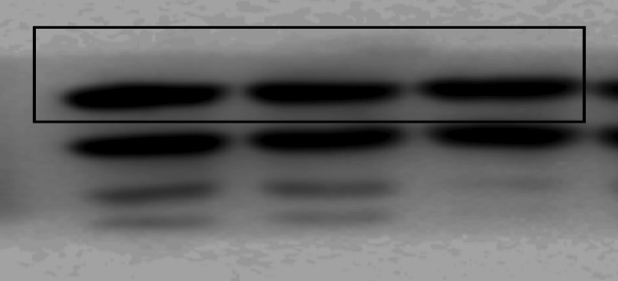


GAPDH-Figure 5C↓


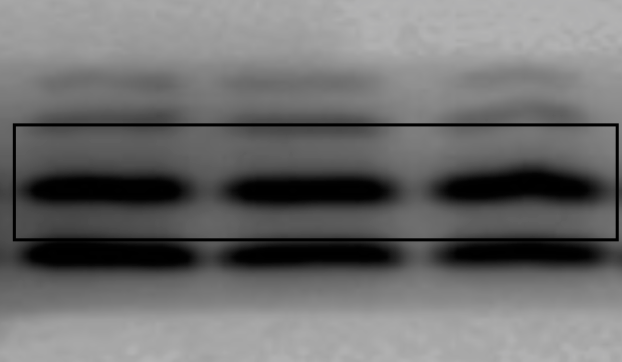


GAPDH-Figure 5E↓


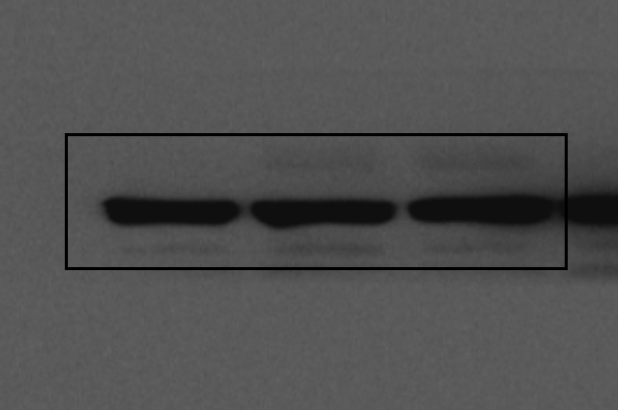


***The unprocessed original scans of western blot for Figure 1C.***

StAR ↓


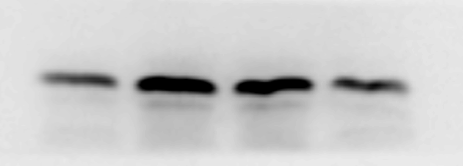


HSD3B2 ↓


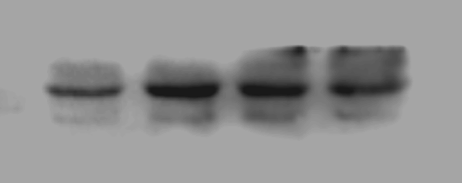


Aromatase ↓


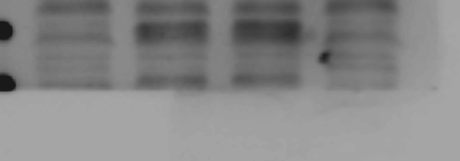


HSD17B1 ↓


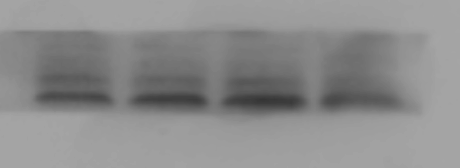


GAPDH ↓


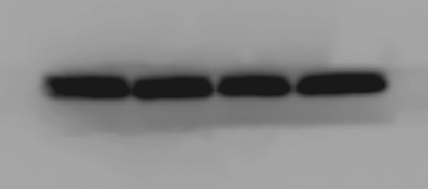


***The unprocessed original scans of western blot for Figure 1E.***

SF-1 ↓


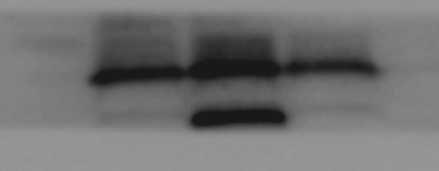


GAPDH ↓


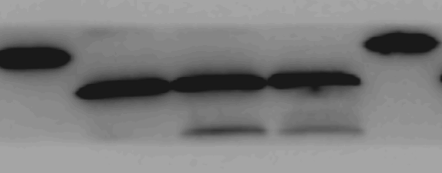


***The unprocessed original scans of western blot for Figure 1F.***

p-CREB↓


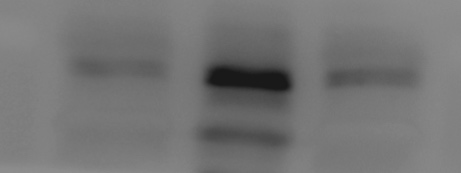


CREB↓


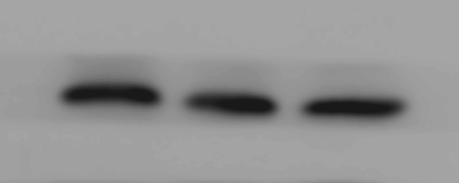


***The unprocessed original scans of western blot for Figure 2A.***

p-p65↓


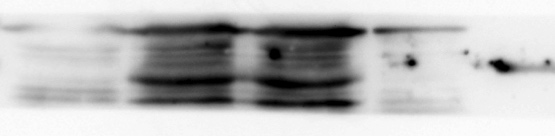


p65↓


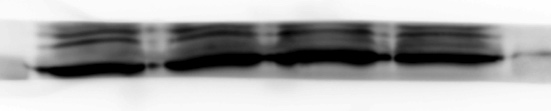


GAPDH↓


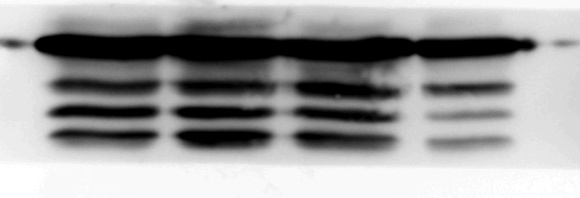


***The unprocessed original scans of western blot for Figure 2D.***

HIF-1α↓


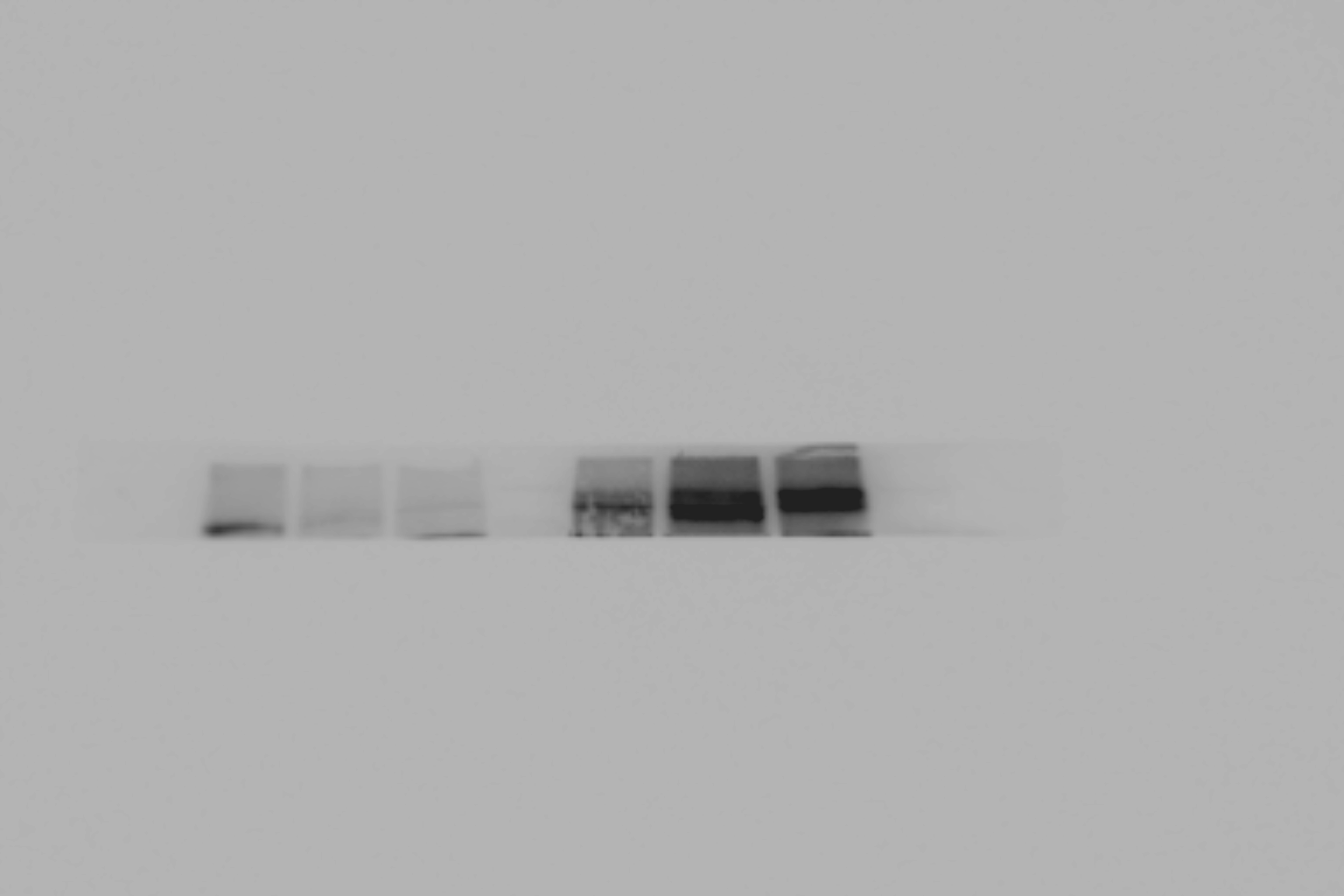


COX-2↓


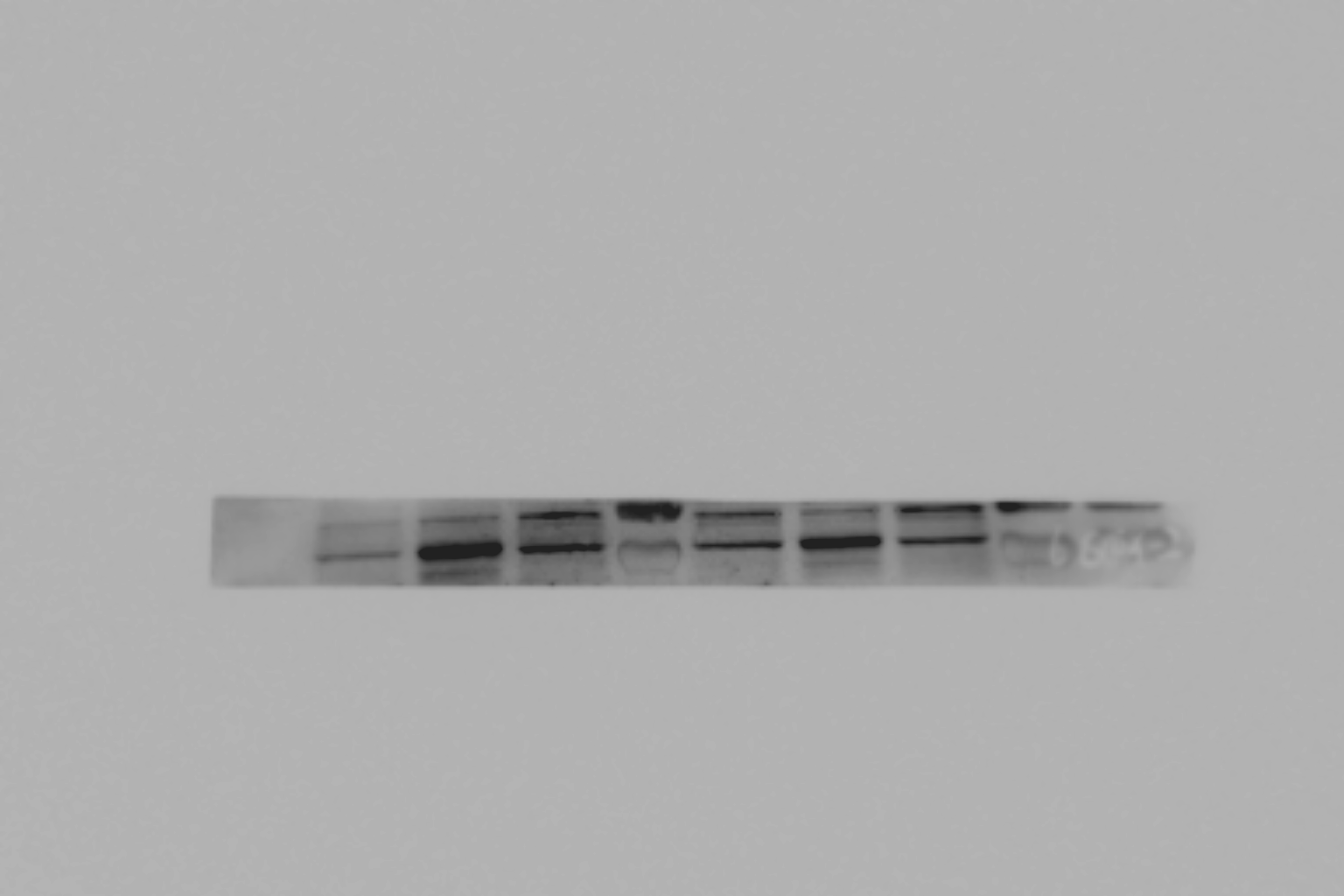


VEGF↓


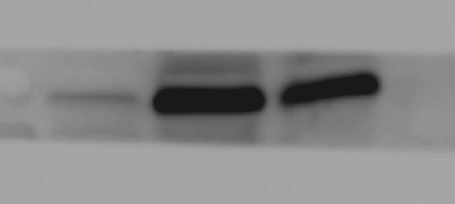


GAPDH↓


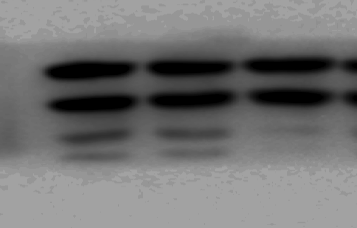


***The unprocessed original scans of western blot for Figure 3C.***

StAR↓


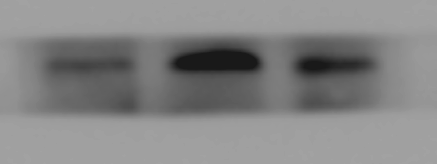


HSD3B2↓


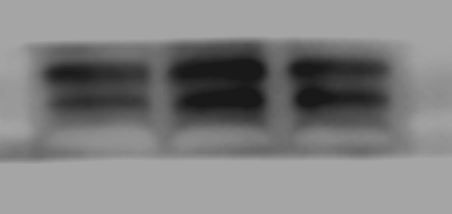


Aromatase↓


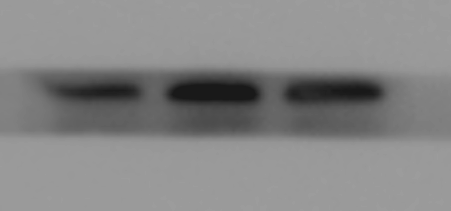


HSD17B1↓


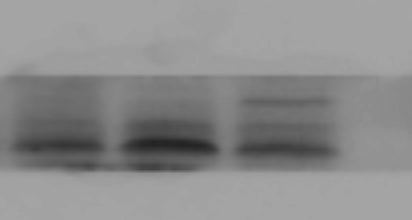


GAPDH↓


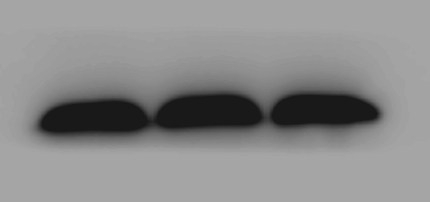


***The unprocessed original scans of western blot for Figure 3E.***

SF-1↓


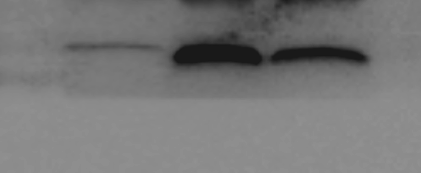


GAPDH↓


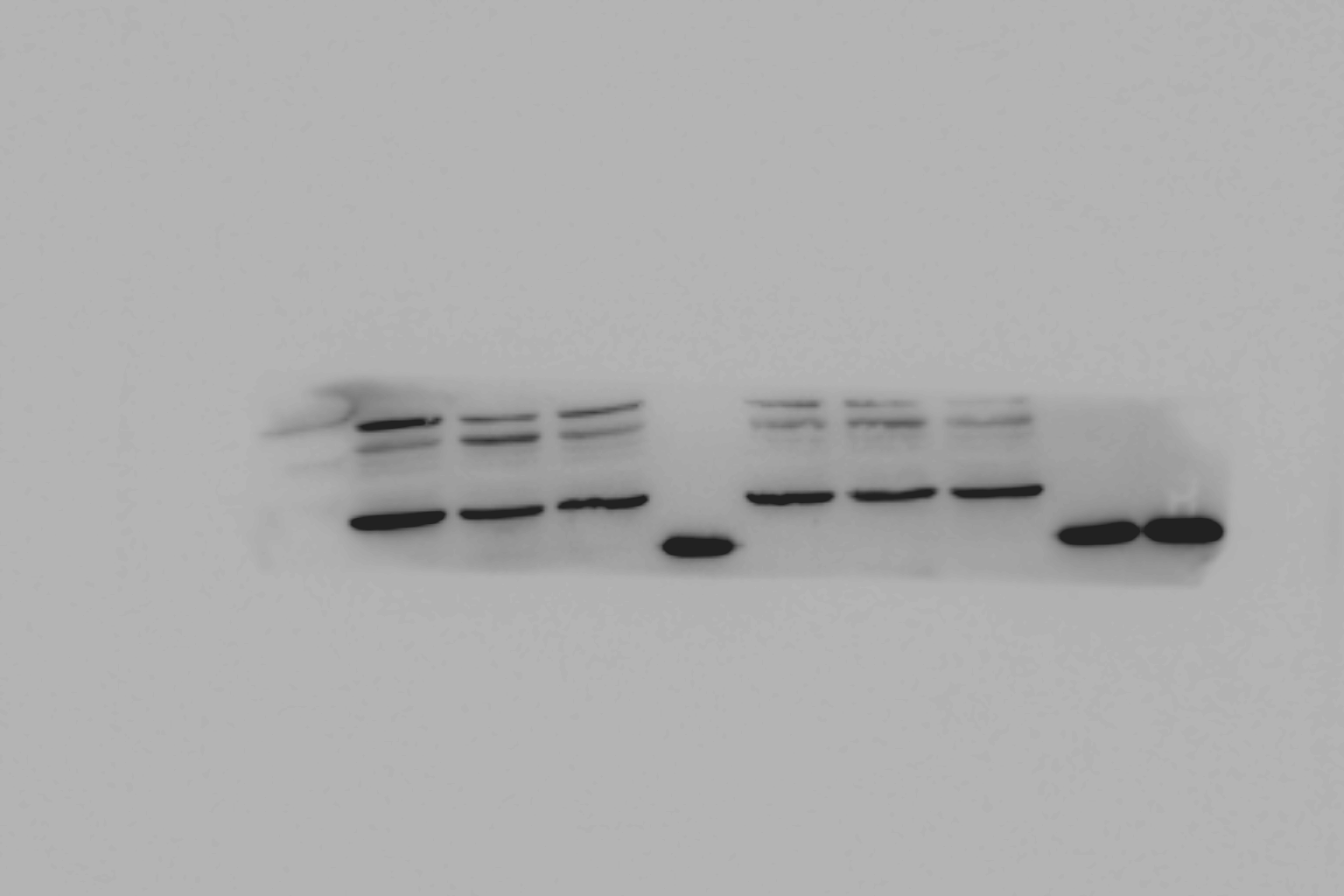


***The unprocessed original scans of western blot for Figure 3F.***

p-CREB↓


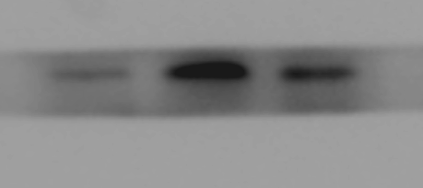


CREB↓


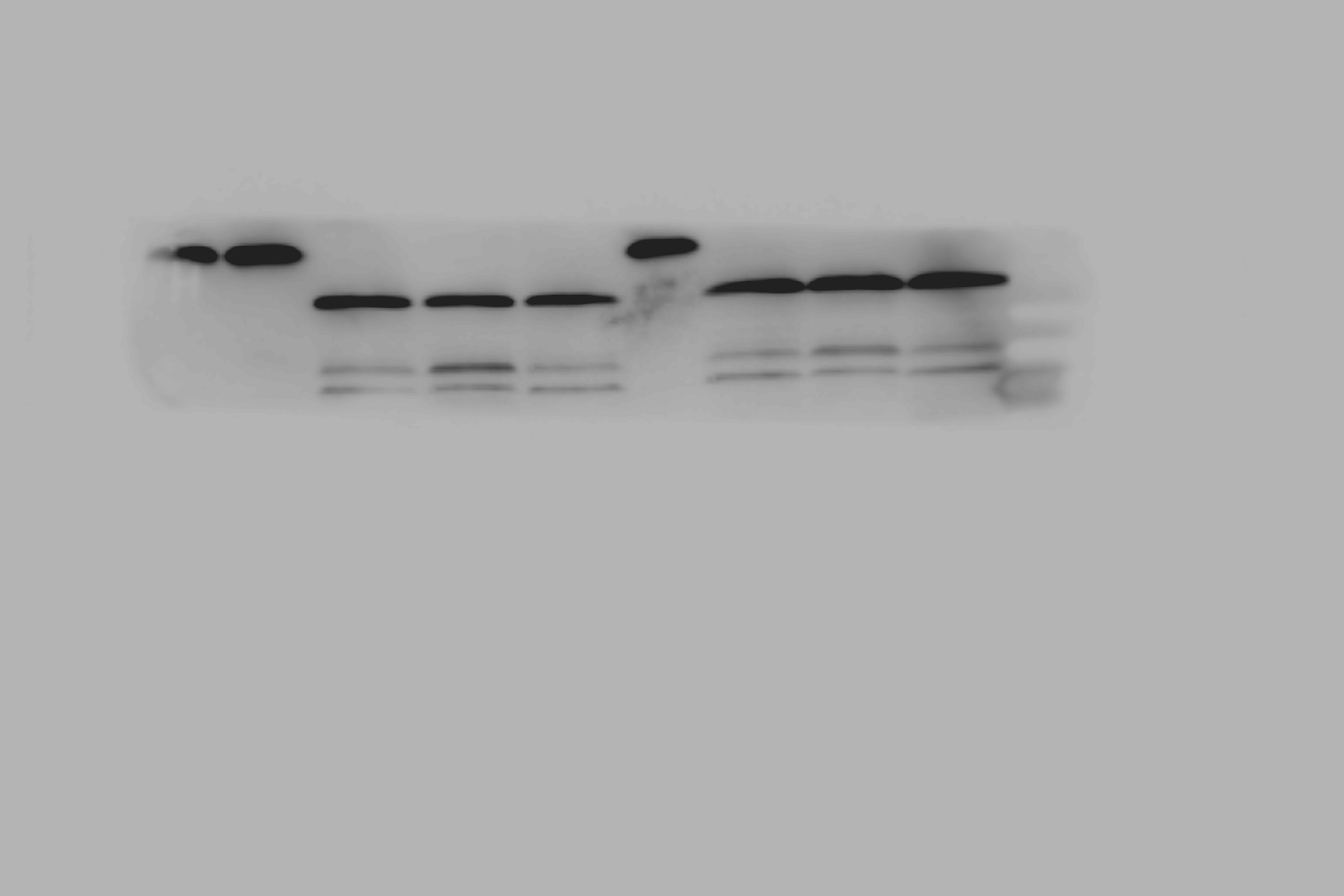


***The unprocessed original scans of western blot for Figure 4B.***

HIF-1α↓


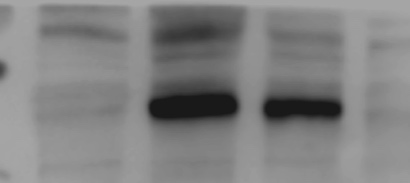


COX-2↓


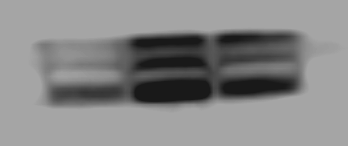


VEGF↓


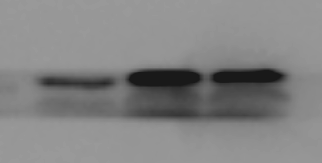


GAPDH↓





***The unprocessed original scans of western blot for Figure 5C***.

StAR↓


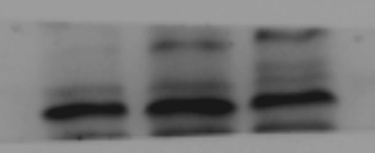


HSD3B2↓


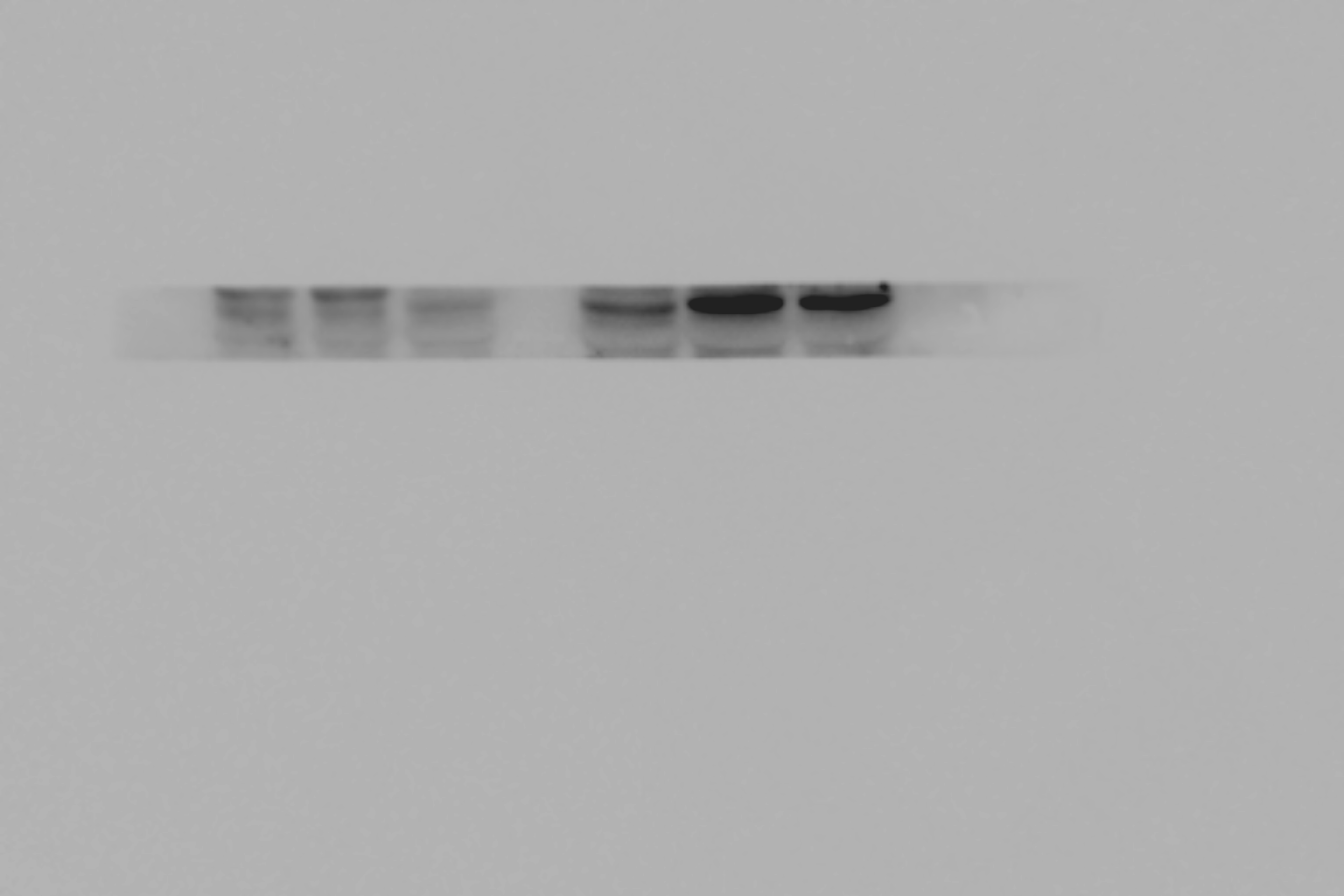


Aromatase↓


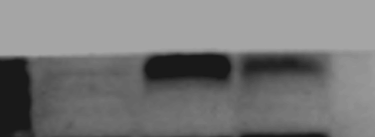


HSD17B1↓


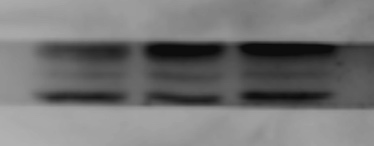


GAPDH↓


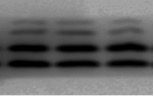


***The unprocessed original scans of western blot for Figure 5E.***

SF-1 ↓


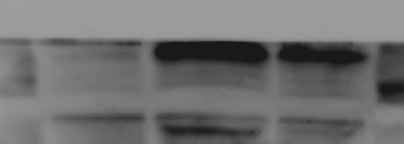


GAPDH↓


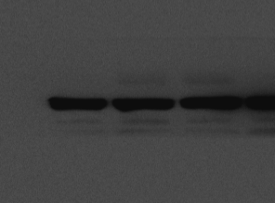


***The unprocessed original scans of Western blot for Figure 5F.***

p-CREB↓


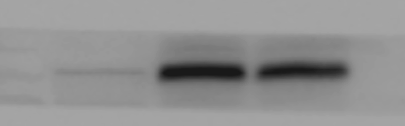


CREB↓


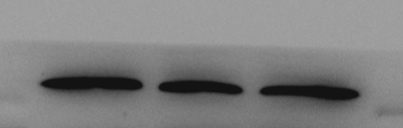

Supplement: Supplementary file 1 — Supplementary information. [file 41598_2020_57997_MOESM1_ESM.docx]
